# IRAP deficiency preserves renal function and attenuates pathology in aged mice

**DOI:** 10.1042/CS20261576

**Published:** 2026-09-16

**Authors:** Sarah L. Walton, Aneesa Ansari, Katrina M. Mirabito Colafella, Lucinda M. Hilliard Krause, Giannie Barsha, Michelle M. Kett, Siew Yeen Chai, Vesna D. Garovic, Kate M. Denton

**Affiliations:** 1 Department of Physiology, Monash University, Clayton, Victoria, Australia; 2 Cardiovascular Disease Program, Monash Biomedicine Discovery Institute, Monash University, Clayton, Victoria, Australia; 3 Neuroscience Program, Monash Biomedicine Discovery Institute, Monash University, Clayton, Victoria, Australia; 4 Department of Obstetrics and Gynecology, Mayo Clinic, Rochester, U.S.A.; 5 Division of Nephrology and Hypertension, Mayo Clinic, Rochester, U.S.A.; 6 Faculty of Medicine, Dentistry and Health Sciences, University of Melbourne, Parkville, Victoria, Australia

**Keywords:** aging, fibrosis, insulin-regulated aminopeptidase, renal physiology, renin-angiotensin system, senescence

## Abstract

Senescent cell accumulation and fibrosis play a fundamental role in kidney ageing. Our aim was to determine whether the angiotensin IV/insulin-regulated aminopeptidase (IRAP) axis of the renin-angiotensin system contributes to renal function decline, fibrosis, and senescent cell accumulation in aged mice. We studied the role of IRAP in kidney ageing using IRAP knockout (KO) mice, as well as in wild-type (WT) mice treated with an IRAP inhibitor. Complementary proximal tubule and collecting duct cell models were used to determine the mechanisms by which IRAP inhibition exerts protective effects. Glomerular filtration rate (GFR) declined by 25% in WT mice between 3 and 23 months of age, concomitant with marked increases in albumin excretion, glomerulosclerosis, tubulointerstitial fibrosis, and accumulation of senescent cells in the proximal tubules of the kidney. IRAP KO mice were protected against age-related decline in GFR, renal fibrosis, and senescence burden compared with age-matched WT mice. IRAP inhibition for 4 weeks (HFI-419, 500 ng/kg/min) in 28-month-old WT mice similarly prevented age-related decline in GFR and attenuated expression of cellular senescence markers. Using an *in vitro* model of cultured proximal tubule cells, IRAP inhibition mitigated the pro-senescent effects of oxidative stress. This was associated with shifts in the metabolic phenotype of proximal tubular cells, indicating the senoprotective effects of IRAP inhibition may be mediated in part via mitochondrial function. These studies provide evidence that IRAP deficiency is protective against chronic renal cellular senescence, fibrosis, and functional decline. Therefore, IRAP inhibition has therapeutic potential to attenuate pathological cardiorenal ageing through senoprotective mechanisms.

## Introduction

The renin-angiotensin system (RAS) plays a central role in cardiovascular ageing and disease. Chronic activation of angiotensin (Ang) II signalling via the angiotensin type I receptor promotes oxidative stress, senescence, inflammation, and fibrosis within the kidney [1,2]. The benefits of RAS blockade, a cornerstone therapy for cardiovascular and renal disease, are attributed in part to attenuation of pro-fibrotic, inflammatory, and oxidative stress pathways [1,2]. Ang IV, a downstream metabolite of Ang II, putatively exerts cardioprotective effects through competitive inhibition of insulin-regulated aminopeptidase (IRAP) [3–5]. Although Ang IV signalling has largely been examined in the context of vascular function, increasing evidence indicates that IRAP regulates hormonal processing and cellular stress pathways that contribute to cardiovascular homeostasis. IRAP is highly expressed in the kidney and participates in diverse biological processes including oxytocin and vasopressin degradation [6,7], antigen cross-presentation [8], and insulin-responsive glucose transport [8]. The distinct cell-specific localisation of IRAP across nephron segments suggests substantial functional heterogeneity [9]. In proximal tubules, IRAP is enriched at the luminal brush borders, consistent with a role in generating free amino acids for reabsorption [9]. In the collecting duct, we and others have shown that IRAP regulates fluid balance through vasopressin degradation and vasopressin V2 receptor/aquaporin 2-mediated signalling [9–14]. Further, IRAP contributes to maternal cardiovascular adaptations to pregnancy in mice [14]. However, the contribution of IRAP to age-related kidney dysfunction is poorly defined.

Several biological processes regulated by IRAP, including insulin-responsive glucose transport and peptide hormone metabolism, are implicated in cellular senescence pathways and age-related cardiorenal dysfunction. As Ang IV exerts many biological effects through IRAP inhibition, these observations raise the possibility that IRAP may represent a previously unrecognised mediator of cellular senescence and organ ageing. Senescence, a cell fate characterised by irreversible permanent growth arrest, altered cell morphology, and altered transcriptome [15], is increasingly recognised as a key driver of cardiorenal ageing. Chronic accumulation of senescent cells promotes organ dysfunction through a complex cascade of inflammation, mitochondrial dysfunction, extracellular matrix accumulation, and fibrosis [16–18]. The kidney is particularly vulnerable due to the high metabolic demand of tubular transport and sustained exposure to oxidative and haemodynamic stress [1,16,19,20]. Moreover, senescence-associated paracrine signalling means relatively few senescent cells can exert systemic effects [21,22]. Although targeted removal of senescent cells has emerged as a promising protective strategy in ageing, these approaches remain in early development [21,23].

Collectively, these observations led us to hypothesise that IRAP accelerates renal ageing by promoting tubular senescence. To test this, we determined whether congenital IRAP deficiency modifies age-related declines in renal function and pathology in mice. We then examined pharmacological IRAP inhibition in aged wild-type (WT) mice to assess whether therapeutic targeting of IRAP could replicate any protective effects observed in IRAP-deficient animals. Complementary *in vitro* models of tubular senescence were examined to elucidate the contributions of IRAP to cellular stress responses.

## Methods

All animal experiments were approved by the Monash Animal Ethics Committee (approval #MARP/2015/037). Experiments were conducted at Monash University (Clayton campus, VIC, 3800 Australia) and conducted in accordance with the Australian Code of Practice for the Care and Use of Animals for Scientific Purposes and NIH Guide for the Care and Use of Laboratory Animals. Mice were housed in standard room conditions (25°C; 12-h light–dark cycle) with *ad libitum* access to a sodium-controlled diet (0.26% wt/wt NaCl) and water.

### Design

#### Study 1: Effect of global IRAP gene deletion

Male and female WT and IRAP knockout (KO) C57BL/6J mice were generated as previously described [24] and maintained at Monash Animal Research Platform. Transcutaneous glomerular filtration rate (GFR) measurements and blood pressure (BP) recordings via radiotelemetry were performed in WT and IRAP KO mice at 3, 15, and 23 months of age. Twenty-four-hour urine collections were assessed in mice at 3 and 23 months of age. Mice were killed by isoflurane overdose (5% isoflurane v/v O_2_) and exsanguination via cardiac puncture at 3 or 23 months of age, in accordance with institutional and international ethical standards. The left kidneys were fixed in 10% neutral buffered formalin and embedded in paraffin, or a commercial fixative (1% glutaraldehyde, 5% methanol in formaldehyde; #9860, Cell Signaling Technologies, MA, U.S.A.) for detection of senescence-associated beta-galactosidase (SA-β-gal). Right kidneys were snap-frozen for molecular analyses.

#### Study 2: Effect of IRAP inhibition

Male C57BL/6J mice were obtained from Monash Animal Research Platform at 3 and 27 months of age. Mice were anaesthetised (2%–2.5% isoflurane v/v O_2_) and implanted with subcutaneous osmotic minipumps (Model #2004, Alzet, CA, U.S.A.) containing vehicle (30% 2-hydroxypropyl-beta cyclodextrin in 5% DMSO) or the IRAP inhibitor, HFI-419 (500 ng/kg/min) for delivery over 28 days. Animals were given analgesia (carprofen, 5 mg/kg in saline s.c.) and bupivacaine (4 mg/kg in saline s.c.) at the incision site at time of surgery, and an additional dose of analgesia (carprofen, 5 mg/kg in saline s.c.) later the same day. Transcutaneous GFR was measured before and at the end of treatment, and a 24 h urine sample was collected at the end of the study. Mice were killed by isoflurane overdose (5% isoflurane v/v O_2_) and exsanguination via cardiac puncture, in accordance with institutional and international ethical standards. Kidneys were fixed and frozen as described in Study 1.

### Radiotelemetry

Male mice were anaesthetised (isoflurane; 2%–2.5% v/v O_2_), and a radiotelemetry probe (PA-C10, Data Sciences International, MN, U.S.A.) was implanted into the left carotid artery for the measurement of arterial pressure (systolic, diastolic, and mean), heart rate, and activity as previously described [25]. Animals were given analgesia (carprofen, 5 mg/kg in saline s.c.) and bupivacaine (4 mg/kg in saline s.c.) at the incision site at time of surgery, and an additional dose of analgesia (carprofen, 5 mg/kg in saline s.c.) later the same day. After a 10-day recovery period, baseline arterial pressure was recorded for 10 s every 10 min over three days using Dataquest ART data acquisition software (Data Science International, MN, U.S.A.).

### Transcutaneous measurement of FITC-sinistrin clearance

Baseline GFR in male and female mice was determined via the transcutaneous clearance of fluorescein isothiocyanate (FITC)-labelled sinistrin using a non-invasive clearance (NIC)-kidney fluorescent detection device (MediBeacon, MO, U.S.A.) [26]. In brief, mice were lightly anaesthetised (2%–2.5% isoflurane v/v O_2_) and a small patch of skin on the mid-back was depilated. The NIC-kidney device was attached to the depilated region using adhesive tape. Following a 1–2-min baseline recording, a bolus of FITC-sinistrin (10–14 mg/100 g body weight, in 0.9% sodium chloride) was administered via the tail vein. Mice were returned to their home cage and elimination kinetics (half-life, *t*^1/2^) were recorded for 1.5 h. GFR was calculated using the two-compartment model (MPD Studio, Medibeacon, Mannheim, Germany) [26].

### Urinalysis

Mice were placed in individual glass metabolic cages for 24 h to collect urine samples (Study 1: 3 and 23 months of age; Study 2: 3 and 28 months of age). All mice were acclimatised to the cages prior to beginning urine collection. Urine samples were collected and stored at −20°C for further analysis. Urinary excretion of albumin and P21, a tubular senescence marker, was determined in duplicate using commercially available mouse ELISA kits (Albuwell M, Exocell, PHL, U.S.A.; ab212072, CAM, U.K., respectively). All urinary parameters were adjusted for urine volume to determine 24-h excretion.

### Kidney fibrosis and glomerulosclerosis

Tubulointerstitial fibrosis (% positivity) and the perivascular fibrosis index (ratio of perivascular fibrosis area to lumen size) were quantified using Aperio ImageScope software with 5 μm paraffin sections stained with Masson’s Trichrome [27,28]. Frozen kidney samples were lyophilised to dry weight, hydrolysed in 6 M hydrochloric acid, and assayed for hydroxyproline (collagen marker) content [29]. Hydroxyproline content was expressed relative to dry tissue weight. In 5 μm sections stained with periodic acid-Schiff (PAS), 30 glomeruli were randomly selected to evaluate glomerulosclerosis using a semi-quantitative method [27]. Glomerulosclerosis was defined as glomerular basement membrane thickening, mesangial hypertrophy, and capillary occlusion, and scored based on pathology: grade 0 (histologically unremarkable), grade 1 (minimal), grade 2 (moderate), grade 3 (moderate-to-severe), and grade 4 (severe).

### Senescence-associated β-galactosidase staining

Following brief fixation in SA-β-gal fixative, kidneys were cryoprotected in 30% sucrose, frozen in Tissue-Tek Optimal Cutting Temperature compound (#4583, Sakura, CA, U.S.A.) and sectioned at 4 μm. Sections were stained for SA-β-gal, a biomarker of cellular senescence [30], using a commercial kit (#9860, Cell Signaling Technology, MA, U.S.A.). Sections were counterstained with nuclear fast red, and positive SA-β-gal staining in the renal cortices was quantified using Aperio ImageScope software as described previously [31].

### IRAP and p21 immunostaining

Paraffin kidney sections (5 μm) were dewaxed, rehydrated, and subjected to heat-mediated antigen retrieval (IRAP: citrate buffer, pH 6.0, 98°C for 20 min; p21: Tris-EDTA buffer, pH 9.0, 98°C for 30 min). Sections were blocked in 10% goat serum for 1 h at room temperature, then incubated in rabbit anti-IRAP antibody (# 6918; Cell Signaling Technology, MA, U.S.A.) or rabbit anti-p21 antibody (#ab188224, Abcam, CAM, U.K.) overnight at 4°C. Slides were washed thoroughly and incubated in a biotinylated anti-rabbit secondary antibody (1:200 dilution; Vector Laboratories, CA, U.S.A.) for 45 min at room temperature, washed, and incubated in avidin–biotin complex (Vector Laboratories) for 30 min. Colour was developed via 3,3′-diaminobenzidine, slides were counterstained with haematoxylin, and scanned. The proportion of IRAP immunostaining within the renal cortex was quantified using Aperio ImageScope software. Automated p21-positive (p21^+^) cell counts were performed using QuPath software (v0.7.0) across six randomly selected regions of interest (0.25 mm^2^) within each renal cortex sample. p21^+^ cells were expressed relative to the total number of nuclei (haematoxylin-positive).

### Quantitative real-time PCR

RNA was extracted from kidney samples using the RNeasy Minikit (Qiagen, VIC, Australia) or from cells using Qiazol (Qiagen, VIC, Australia). All RNA was treated with deoxyribonuclease I and assessed for quantity and purity using a spectrophotometer. One microgram of RNA was reverse transcribed to cDNA (iScript™ Supermix, BioRad, NSW, Australia). Amplification was performed using Taqman reagents (Life Technologies, VIC, Australia) with 25 ng cDNA per well [32]. Taqman gene expression assay codes are listed in Supplementary Table S1. The comparative cycle threshold method was used for all expression assays using 18s as the endogenous control.

## *In vitro* studies on immortalised kidney cell lines

Madin–Darby canine kidney (MDCK) cells and human renal proximal tubular epithelial cells (HK-2; source ATCC, Manassas, VA, U.S.A.) were cultured in DMEM and DMEM-F12, respectively, supplemented with 10% fetal bovine serum, 1% penicillin, and 1% streptomycin. Cells were grown in 5% CO_2_ at 37°C in a humidified incubator. Preliminary dose–response studies were performed to determine optimal doses of H_2_O_2_ to induce ∼50%–60% cellular senescence in MDCK and HK-2 cells. Cells were seeded in 96-well plates and treated for 24 h with H_2_O_2_ (MDCK: 1300 μM; HK-2: 800 μM) to induce cellular senescence, in the presence or absence of HFI-419 (0.01 μM). DMSO was used as a vehicle control for all experiments. Data were obtained from three experiments, with three technical replicates within each experiment.

### Detection of IRAP via western blotting

MDCK and HK-2 cells were washed with ice-cold phosphate-buffered saline (pH 7.4) solution and lysed in radioimmunoprecipitation assay buffer supplemented with a protease/phosphatase inhibitor cocktail (#5872, Cell Signaling, MA, U.S.A.). Lysates were centrifuged at 13,000 rpm at 4°C for 10 min. Total protein content was determined using a protein assay kit (#23200, Thermo Fisher Scientific, VIC, Australia). Samples were prepared using LDS sample buffer (#B0007, Thermo Fisher Scientific, VIC, Australia) and reducing reagent (#B0004, Thermo Fisher Scientific, VIC, Australia) then heat-treated at 70°C for 10 min. Protein was resolved via electrophoresis using precast 4%–12% Bis–Tris gels (Thermo Fisher Scientific, VIC, Australia) and transferred onto a nitrocellulose membrane. The membrane was washed and blocked for 30 min using 5% BSA and 0.1% TBST (10 mM Tris−HCl, 150 mM NaCl, 0.1% Tween 20). Following this, the membrane was incubated overnight at 4°C in antibodies against IRAP (1:2000 dilution; # 6918, Cell Signaling Technology, MA, U.S.A.) and β-actin (1:2000 dilution; #4970, Cell Signaling Technology, MA, U.S.A.). The membrane was washed and incubated with anti-rabbit peroxidase-conjugated secondary antibodies (Cell Signaling, MA, U.S.A.) for 1 h. Protein bands were visualised by enhanced chemiluminescence (Roche, Basel, Switzerland) and a ChemiDoc MP system (Bio-Rad, CA, U.S.A.).

### Cell viability assay

Cells were seeded in 96-well plates (1 × 10^4^ cells/well) and subjected to treatments as described above. Cell viability was assessed using an MTS assay kit (ab#197010, Abcam, U.K.). Absorbance was measured at 490 nm, and data were expressed as a percentage of the vehicle control.

### Senescence-associated β-galactosidase staining

Following treatment with H_2_O_2_ in the presence and absence of HFI-419, cells were fixed and stained for SA-β-gal activity, as described for *in vivo* studies. Three fields of view (×20 magnification) per well were captured for three independent experiments. SA-β-gal-positive cells were counted and expressed as a proportion of total cell number.

### Cell migration assay

Cells were plated in a six-well plate (5 × 10^4^ cells/well) and allowed to adhere overnight. A uniform scratch was made using a 1 ml pipette tip on the day of the experiment. Cells were treated with H_2_O_2_ in the presence and absence of HFI-419, as described above. Live cell images were captured using a confocal microscope (Leica DMIi, Monash MicroImaging Platform) at 10× magnification every 2 h for up to 48 h to monitor scratch closure.

### XF real-time ATP rate assay

ATP production rates were assessed using the XFe24 Seahorse analyser and XFp Real-Time ATP Rate Assay kit (Agilent Technologies). On the day prior to the experiment, MDCK (5 × 10^4^/well) and HK-2 cells (3 × 10^4^/well) were seeded in XF24 cell culture microplates coated with entactin-collagen IV-laminin cell attachment matrix (#631947, Merck, Germany) in complete media. Cells were incubated for 4 h with media containing H_2_O_2_, and/or HFI-419. Culture media was replaced with XF Assay Medium (Agilent Technologies, Canada) and plates were assayed in the XF24 analyser. Basal oxygen consumption rate (OCR), a measurement of mitochondrial respiration, and extracellular acidification rate (ECAR), a measurement of glycolysis, were determined at injection of oligomycin (ATP synthase blocker; 1.5 μM) to determine ATP turnover and proton leak. Rotenone plus antimycin A (Rot/AA; inhibitors of complexes I and III of the electron transport chain; 0.5 μM) was then injected to determine mitochondrial-associated acidification. OCR and ECAR rates were normalised to cell counts following DAPI staining.

### Statistics

All statistical data are presented as mean ± standard error of mean (SEM). A two-way ANOVA with the factors genotype and age was used to analyse all Study 1 data and Study 2 GFR data. Specific comparisons within groups were performed via a Sidak’s post-hoc analysis test. Remaining Study 2 data and all *in vitro* data were analysed by one-way ANOVA followed by a Sidak’s test for multi-group comparisons. A *P*-value of *α* < 0.05 was considered statistically significant.

## Results

### IRAP deficiency prevents age-related decline in renal function

**Figure 1:**
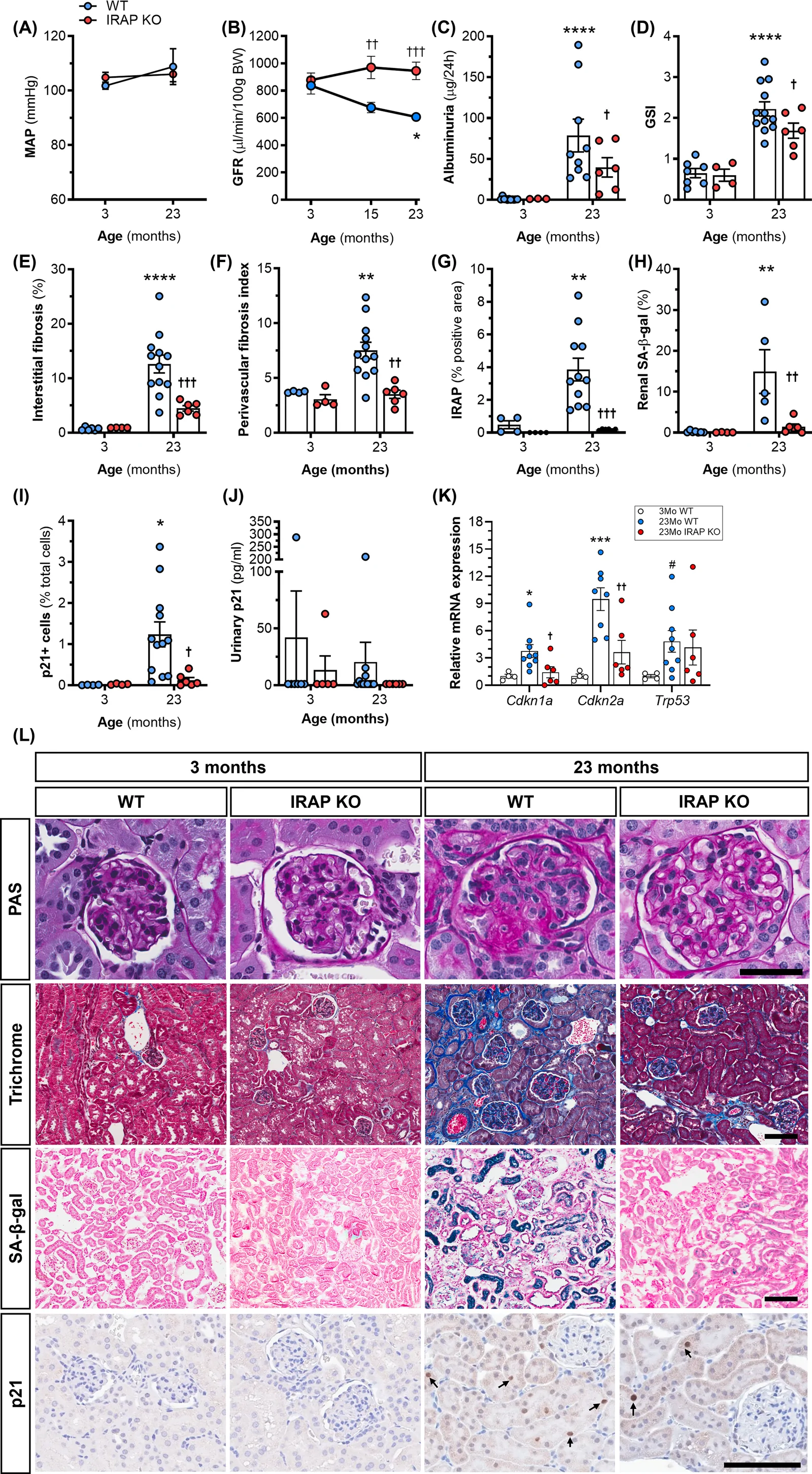
IRAP deficiency alleviates cellular senescence and prevents decline in renal function in ageing male mice (**A**) Mean arterial pressure, (**B**) GFR, (**C**) albuminuria, (**D**) glomerulosclerosis index (GSI), (**E**) renal interstitial fibrosis quantified via Masson’s trichrome staining, (**F**) perivascular fibrosis index (ratio of perivascular cross-sectional area to lumen cross-sectional area), (**G**) renal IRAP immunostaining positivity, (**H**) renal SA-β-gal, (**I**) nuclear p21 positivity (+), expressed as a proportion of total nuclei, and (**J**) urinary p21 excretion in male WT (red) and IRAP KO (blue) mice from 3 to 23 months of age. (**K**) mRNA expression of p21 (*Cdkn1a*), p^16INK4a^ (*Cdkn2a*), and p53 (*Trp53*), expressed relative to the endogenous control (*18s*). (**L**) Histology panel shows PAS staining to determine GSI, Masson’s trichrome staining to detect tubulointerstitial fibrosis, SA-β-gal staining to detect senescent cells (blue staining), and p21 immunostaining (arrows point to p21+ nuclei). Scale = 50 μm. *n =* 4–10/group. (A–J) Data were analysed via two-way ANOVA followed by a Sidak’s multiple comparisons test; **P<0.01, ****P<0.0001 versus 3-month-old WT; †*P<*0.05, ††*P<*0.01, †††*P<*0.001 versus age-matched WT. Age effects were assessed relative to 3-month-old mice, whereas genotype effects were assessed by comparison with age-matched WT controls. (K) Data were analysed via a one-way ANOVA with Sidak’s multiple comparisons tests comparing 3-month-old WT to 23-month-old WT and 23-month-old WT to 23-month-old IRAP KO; #*P* = 0.06, *P<0.05, **P<0.01, ***P<0.001, ****P<0.0001 versus 3-month-old WT; †*P<*0.05, ††*P<*0.01 versus 23-month-old WT.

To explore the functional role of IRAP in the ageing kidney, we compared BP, renal function, and kidney disease markers in young (3-month-old) and aged (23-month-old) WT and IRAP KO mice. Mean arterial pressure was not different between WT and IRAP KO mice at 3 and 23 months of age (Figure 1A). IRAP deficiency did not affect body or kidney weights of male or female mice (Supplementary Figure S1). From 3 to 23 months of age, GFR declined by ∼27% in male WT mice (*P<*0.05; Figure 1B) and ∼32% in female WT mice (*P<*0.01, Supplementary Figure S2A). This decline in GFR was associated with an ∼80-fold increase in albuminuria in male (*P<*0.0001, Figure 1C) and a ∼37-fold increase in female WT mice (*P<*0.01, Supplementary Figure S2B). Age-related albuminuria and decline in renal function were attenuated in IRAP KO male (Figure 1B,C) and female (Supplementary Figure S2) mice.

### IRAP deficiency prevents age-related increases in renal fibrosis and senescent cell burden

Age-related decline in renal function in WT mice was associated with significant glomerulosclerosis (Figure 1D,L), widespread tubulointerstitial fibrosis (*P<*0.0001, male: Figure 1E,L; female: Supplementary Figure S2D) and perivascular fibrosis (*P<*0.01, Figure 1F). We conducted immunostaining to examine the expression pattern of IRAP in the ageing WT mouse kidney (Figure 1G and Supplementary Figure S3). Immunostaining for IRAP was detected in renal tubular epithelia in all animals (Supplementary Figure S3). High levels of IRAP expression were localised predominantly in the distal tubules and collecting ducts of all animals (Supplementary Figure S3). In aged animals, IRAP expression was also detected in the proximal tubules and glomeruli of the kidney (Supplementary Figure S3). IRAP expression was ∼8-fold greater in the renal cortex of 23-month-old male WT mice than in that of 3-month-old counterparts (*P<*0.01; Figure 1G and Supplementary Figure S3). As expected, IRAP was not expressed in the kidneys of IRAP-deficient mice.

Staining for SA-β-gal revealed marked accumulation of senescent cells within the kidney of the aged male (Figure 1H,L) and female (Supplementary Figure S2C) WT mice. SA-β-gal staining was predominantly localised to the proximal and distal tubules in the renal cortex, with occasional SA-β-gal^+^ cells evident in the glomeruli (Figure 1H,L). Nuclear cyclin-dependent kinase inhibitor (p21) staining was enhanced in aged WT kidneys compared with young WT kidneys (*P<*0.05, Figure 1I,L). Similar to SA-β-gal activity, p21 expression was generally localised to the proximal and distal tubule epithelia of aged WT mice (Figure 1L). IRAP KO mice were protected from age-related increases in glomerulosclerosis, tubulointerstitial fibrosis, perivascular fibrosis, and renal senescent cell burden (male: Figure 1D–F; female: Supplementary Figure S2). Only occasional SA-β-gal-positive and p21-positive cells were detected in kidneys of IRAP KO mice, predominantly in the proximal tubules (Figure 1L). Urinary excretion of p21, a tubular senescence marker, was largely undetectable in mice of any age or genotype (Figure 1J). mRNA expression of key cellular senescence markers *Cdk1na (*p21; *P<*0.05*), Cdk2na* (p16^INK4a^, *P<*0.001) and *Trp53* (p53; *P* = 0.06) was elevated in kidneys from aged WT mice, relative to 3-month-old WT mice (Figure 1K). Expression of senescence markers in kidneys from IRAP KO mice was markedly blunted compared with aged WT mice (Figure 1K), indicating that IRAP deficiency is protective against age-related increases in kidney senescent cell burden.

### Short-term IRAP inhibition prevents age-related decline in renal function in elderly mice

**Figure 2:**
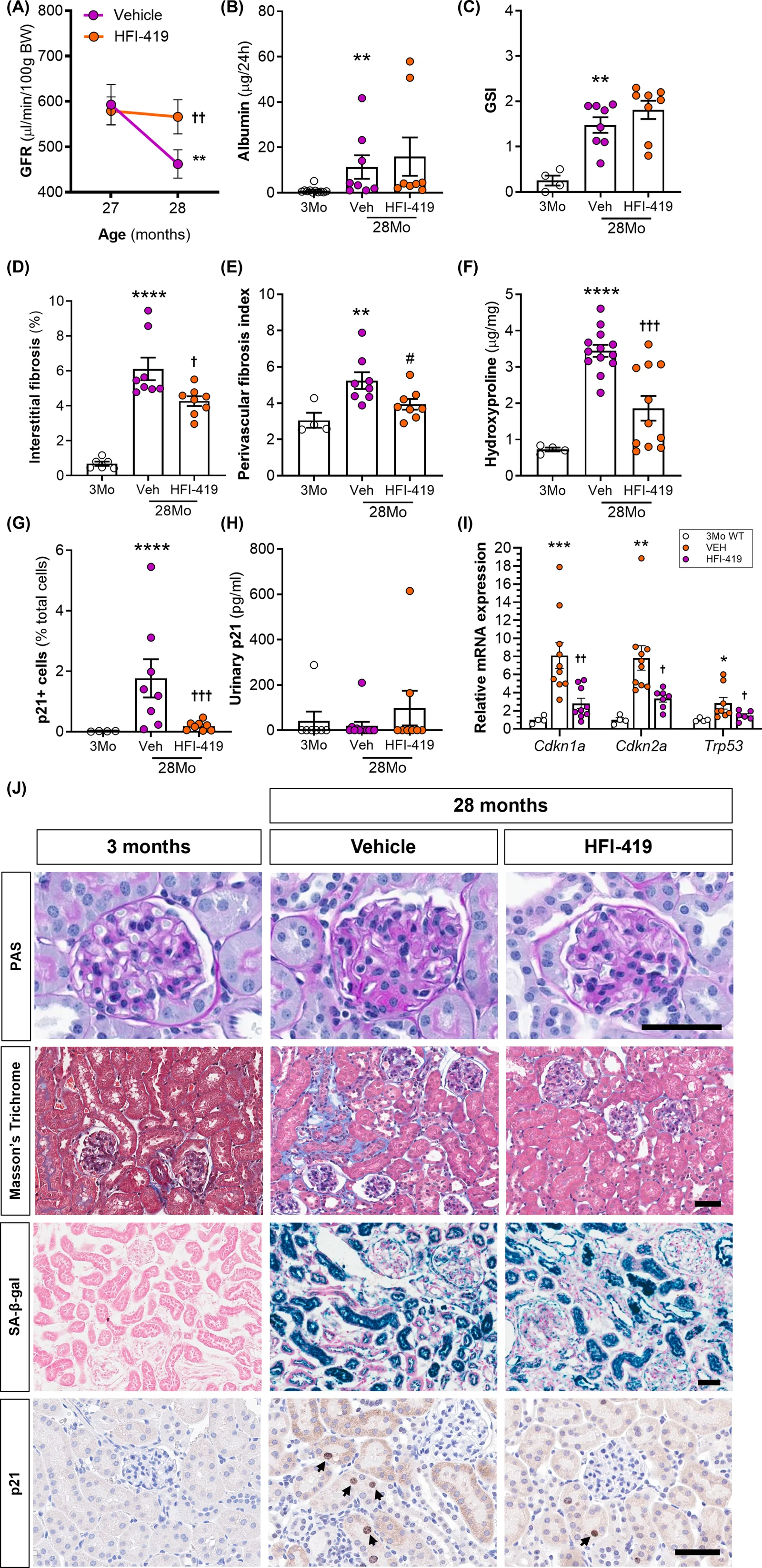
IRAP inhibition alleviates kidney fibrosis and improves kidney function in aged mice (**A**) GFR in male WT mice at 27 months of age, before and after 28 days of vehicle (purple) or HFI-419 (orange) infusion. (**B**) Albuminuria, (**C**) GSI, (**D**) renal interstitial fibrosis quantified via Masson’s trichrome staining, (**E**) perivascular fibrosis index (ratio of perivascular cross-sectional area to lumen cross-sectional area), (**F**) renal hydroxyproline content, (**G**) nuclear p21 positivity (+), expressed as a proportion of total nuclei, (**H**) urinary p21 excretion, and (**I**) renal mRNA expression of p21 (*Cdkn1a*), p16^INK4a^ (*Cdkn2a*), and p53 (*Trp53*). A 3-month-old WT group is shown as a control (open points). (**J**) Histology panel shows PAS staining to determine GSI, Masson’s trichrome staining to detect tubulointerstitial and perivascular fibrosis, SA-β-gal staining to detect senescent cells (blue staining), and p21 immunostaining (arrows point to representative p21-positive cell nuclei). Scale = 50 μm. *n =* 4–10/group. (A) Data were analysed via two-way ANOVA followed by a Sidak’s multiple comparisons test; **P<0.01 versus 27-month-old vehicle; ††*P<*0.01 versus 28-month-old vehicle. Age effects were assessed relative to 27-month-old mice, whereas IRAP inhibition effects were assessed by comparison with age-matched vehicle controls. **P<0.01 versus 27-month-old vehicle; ††*P<*0.01 versus 28-month-old vehicle. (B–I) Data are analysed via one-way ANOVA. *P<0.05, **P<0.01, ***P<0.001, ****P<0.0001 versus 3-month-old WT; #*P* = 0.05, †*P<*0.05, ††*P<*0.01, †††*P<*0.001 versus 28-month-old vehicle via Sidak’s multiple comparisons test. Age effects were assessed by comparing vehicle-treated 3-month-old to 28-month-old WT mice, and IRAP inhibition effects were assessed by comparing 28-month-old vehicle to 28-month-old HFI-419-treated mice.

We next assessed the utility of short-term IRAP inhibition in preventing age-related decline in kidney function in elderly WT C57BL/6 mice using the IRAP inhibitor HFI-419. GFR in vehicle-treated WT mice declined by ∼22% from 27 to 28 months of age (*P<*0.01, Figure 2A). IRAP inhibition for 28 days prevented the age-related decline in GFR observed in vehicle-treated WT mice (*P<*0.01; Figure 2A). Marked increases in albuminuria (11-fold greater; *P<*0.01, Figure 2B), glomerulosclerosis (*P<*0.0001, Figure 2C), renal interstitial fibrosis (nine-fold greater, *P<*0.0001, Figure 2D), perivascular fibrosis (*P<*0.01, Figure 2E), renal hydroxyproline content (a collagen marker, 4.7-fold greater; *P<*0.0001, Figure 2F), and p21+ renal cell burden (*P<*0.001, Figure 2G) were observed in 28-month-old WT mice compared with 3-month-old WT mice. Kidneys from 28-month-old WT mice showed prominent, widespread staining for SA-β-gal, primarily in the proximal tubules but also present in the glomeruli and interstitium (vehicle: 38.4% ± 6.4%; HFI-419: 38.9% ± 4.4%; Figure 2J). We then examined the localisation and expression of p21 as a cellular senescence marker using immunohistochemistry. Minimal p21 nuclear positivity was detected within the renal cortex of young mice, with up-regulation detected in vehicle-treated 28-month-old WT mice (*P<*0.01, Figure 2G,J). IRAP inhibition for 28 days had no effect on albuminuria (Figure 2B) and glomerulosclerosis (Figure 2C) in aged WT mice. However, HFI-419 treatment reduced tubulointerstitial fibrosis (*P<*0.01, Figure 2D) and perivascular fibrosis (*P* = 0.054, Figure 2E) compared with vehicle treatment. This was confirmed by a ∼1.7-fold reduction in renal hydroxyproline content in mice treated with HFI-419 relative to vehicle-treated animals (*P<*0.01, Figure 2F). p21 excretion was unaffected by age or IRAP inhibition (Figure 2H). The kidneys of the IRAP inhibitor-treated mice, similar to those of the vehicle treatment, displayed widespread renal SA-β-gal staining throughout the renal tubules, glomeruli, and interstitium (Figure 2J). Nuclear p21 positivity was blunted in aged mice treated with the IRAP inhibitor compared with vehicles (*P<*0.05, Figure 2G). Renal mRNA expression of cellular senescence markers (*p21, Cdkn2a, Trp53*) was greater in 28-month-old WT mice infused with vehicle compared with 3-month-old WT mice (all *P<*0.05, Figure 2I). IRAP inhibition suppressed mRNA expression of all cellular senescence markers (all *P<*0.05, Figure 2I).

### HFI-419 attenuates cellular senescence and promotes wound healing in stressed proximal tubule epithelial cells

From our *in vivo* studies, we concluded that IRAP contributes to the renal senescence burden, fibrosis, and kidney function decline with ageing. We further explored the mechanisms underlying the role of IRAP in *in vitro* models of kidney tubule senescence using HK-2 and MDCK cells. IRAP protein was detected in both HK-2 and MDCK cell lines at ∼165 kDa (Supplementary Figure S4). HK-2 and MDCK cells were then cultured in the presence or absence of HFI-419 in a pro-senescent milieu of H_2_O_2_ for 24 h. Both cell lines displayed morphological characteristics of senescence, including enlarged and flattened cell bodies, alongside increased SA-β-gal activity in response to H_2_O_2_. Thus, confirming the ability of H_2_O_2_ to induce senescence in renal tubular cells.

**Figure 3:**
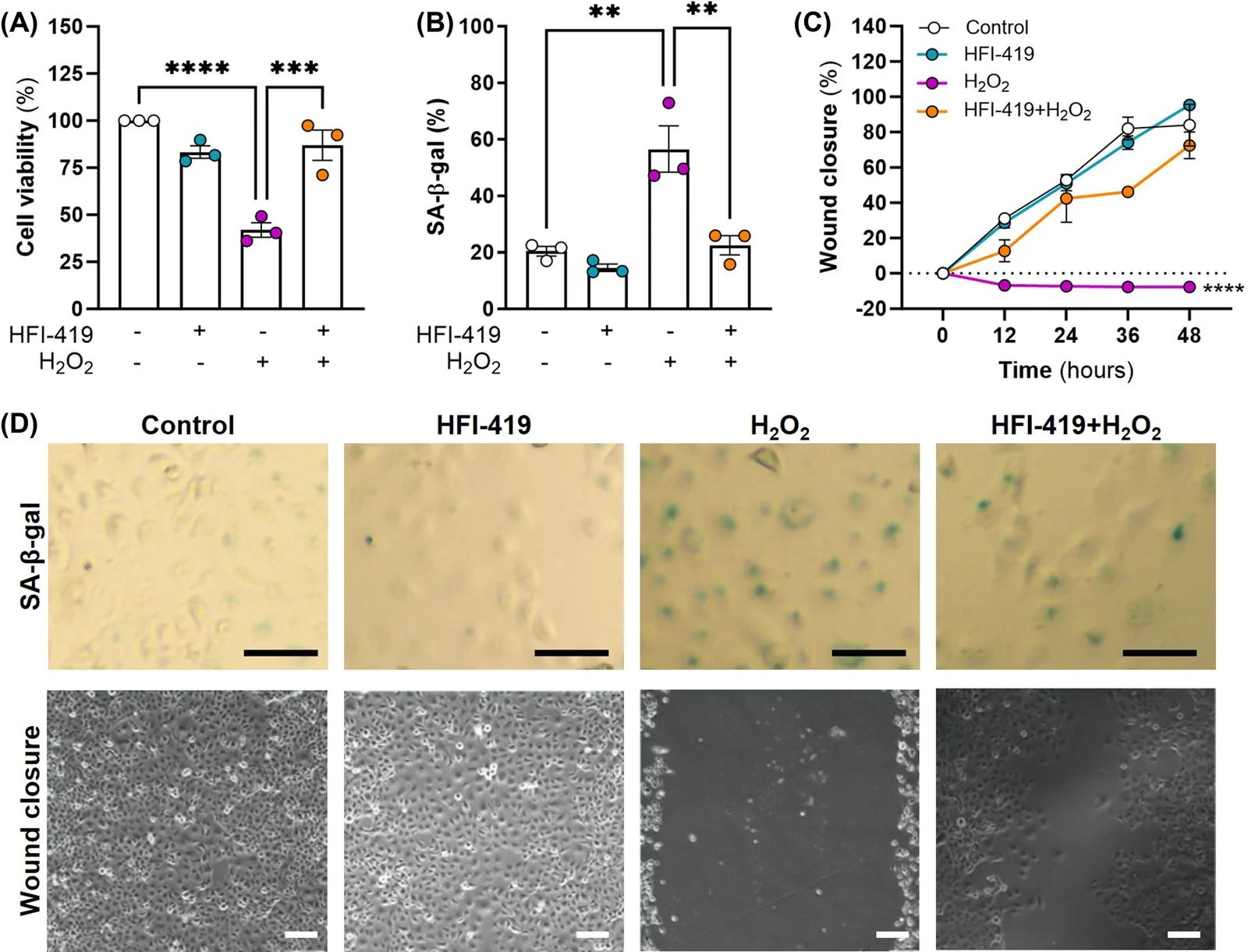
IRAP inhibition attenuates hydrogen peroxide-induced senescence and promotes wound healing in HK-2 cells HK-2 cells were exposed to DMSO (control), HFI-419 (0.01 μM), H_2_O_2_ (800 μM), or H_2_O_2_ ± HFI-419 for 24 h. (**A**) Cell viability measured via an MTS assay. (**B**) SA-β-gal^+^ cells (%) quantified using SA-β-gal staining (**D**, scale = 100 μm). (**C**) Wound closure (%) over 48 h, with representative images at the 48-h timepoint shown in (**D**; scale = 100 μm). Values represent mean ± SEM; *n =* 3 from independent experiments, each performed with three technical replicates. Data analysed via one-way ANOVA with Sidak’s multiple comparisons tests. Pairwise comparisons were control versus H_2_O_2_, control versus HFI-419, H_2_O_2_ versus HFI-419 and control versus HFI-419+ H_2_O_2_; **P<0.01, ***P<0.001, ****P<0.0001.

H_2_O_2_ treatment for 24 h reduced HK-2 cell viability to ∼42% (Figure 3A; *P<*0.0001 versus control) and increased SA-β-gal activity to ∼57% (Figure 3B, D, *P<*0.001 versus control). HFI-419 treatment alone did not affect viability (Figure 3A; *P* = 0.11 versus control) or SA-β-gal activity (Figure 3B; *P* = 0.78 versus control). Treatment with HFI-419 under H_2_O_2_ conditions preserved HK-2 cell viability (Figure 3A) and attenuated SA-β-gal activity (Figure 3B) to levels equivalent to the control. As cellular capacity to respond to wound healing is impaired in senescent cells, we performed a scratch assay to determine whether IRAP inhibition exerts protective effects in H_2_O_2_-induced cellular stress. Wound closure was almost complete (∼95%) within 48 h in control HK-2 cells; this response was severely limited by H_2_O_2_ (*P<*0.0001 versus control; Figure 3C, D). HFI-419 co-treatment significantly enhanced the migratory potential of H_2_O_2_-stressed HK-2 cells (∼72% closure by 48 h; *P<*0.0001 versus H_2_O_2_ treatment; Figure 3C). Together these data indicate that inhibition of IRAP can mitigate, at least partially, the pro-senescent and anti-migratory effects of renal oxidative stress.

### H_2_O_2_ induces cellular senescence in MDCK cells

**Figure 4:**
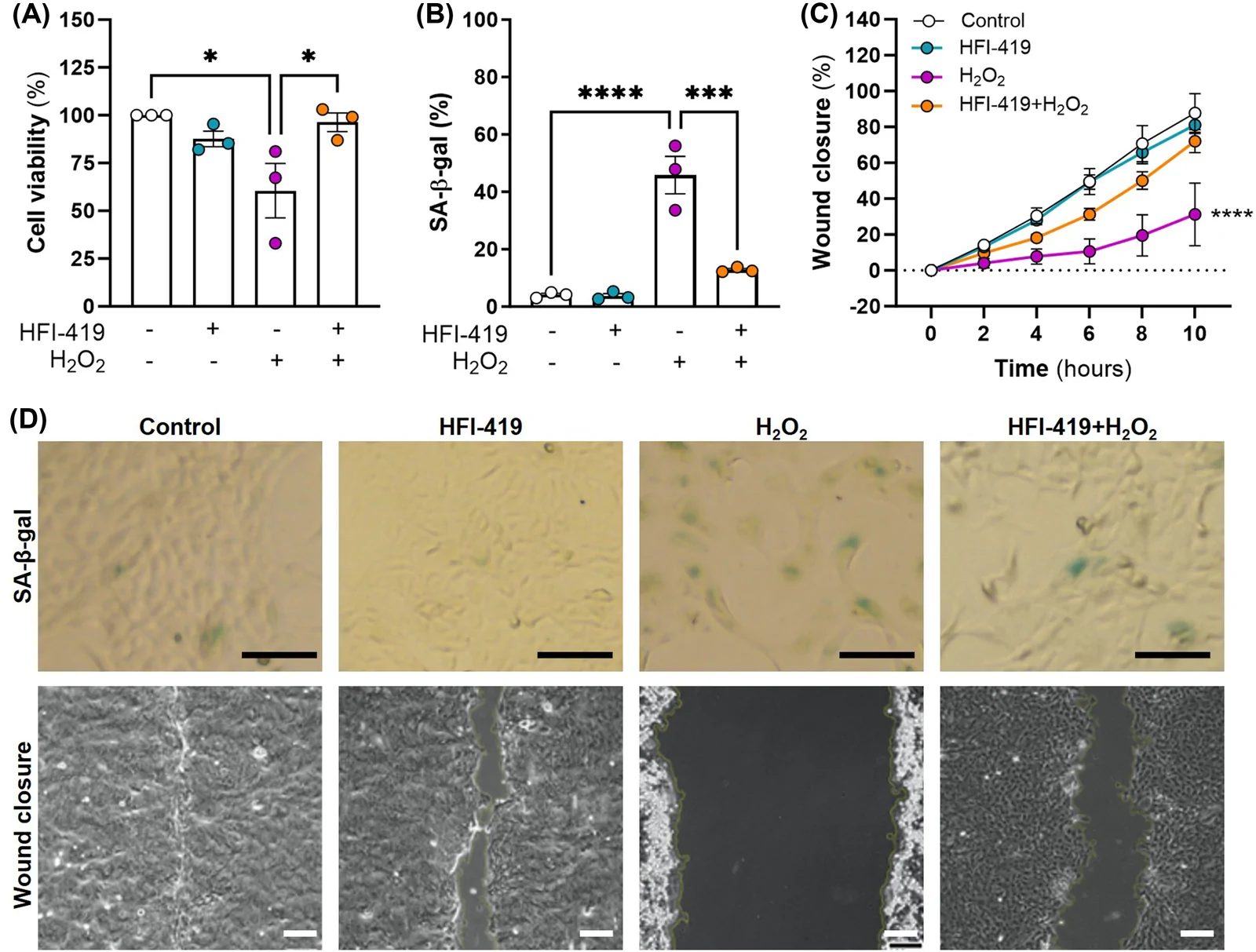
IRAP inhibition attenuates hydrogen peroxide-induced senescence and promotes wound healing in MDCK cells MDCK cells were exposed to DMSO (control), HFI-419 (0.01 μM), H_2_O_2_ (800 μM), or H_2_O_2_ ± HFI-419 for 24 h. (**A**) Cell viability measured via an MTS assay. (**B**) SA-β-gal^+^ cells (%) quantified using SA-β-gal staining (**D**, scale = 100 μm). (**C**) Wound closure (%) over 48 h, with representative images at the 48-h timepoint shown in (D; scale = 100 μm). Values represent mean ± SEM; *n =* 3 from independent experiments, each performed with three technical replicates. Data analysed via one-way ANOVA with Sidak’s multiple comparisons tests. Pairwise comparisons were control versus H_2_O_2_, control versus HFI-419, H_2_O_2_ versus HFI-419 and control versus HFI-419+ H_2_O_2_; *P<0.05, ***P<0.001, ****P<0.0001.

In MDCK cells, H_2_O_2_ treatment for 24 h reduced cell viability (∼60% of control, *P<*0.05 versus control, Figure 4A) and stimulated SA-β-gal activity (∼50% positive cells, *P<*0.0001 versus control, Figure 4B, D). HFI-419 had no effect on MDCK cell viability (Figure 4A, *P* = 0.65 versus control) or SA-β-gal activity (Figure 4B, *P* = 0.99 versus control). Combined treatment of MDCK cells with HFI-419 and H_2_O_2_ improved cellular viability to levels comparable to control (∼96% viability, *P<*0.05, Figure 4A). This was associated with a reduced senescence burden compared with H_2_O_2_ treatment alone (∼13% SA-β-gal positivity, *P<*0.001 versus H_2_O_2_ treatment, Figure 4B). The wound healing assay revealed that untreated MDCK cells achieved wound closure within 10 h (Figure 4C, D). MDCK cells treated with H_2_O_2_ achieved ∼31% wound closure in 10 h, a significant impairment compared with untreated control cells (*P<*0.0001 versus control, Figure 4C). HFI-419 improved the wound healing response in the H_2_O_2_ milieu (∼72% closure within 10 h, *P<*0.001 versus H_2_O_2_-treated cells; Figure 4C). This shows that, like HK-2 cells, MDCK cells are responsive to IRAP inhibition as shown by improvements in cell viability and migratory capacity, and reduced senescence burden in the setting of oxidative stress.

### IRAP inhibition influences mitochondrial respiration in HK-2 cells

**Figure 5:**
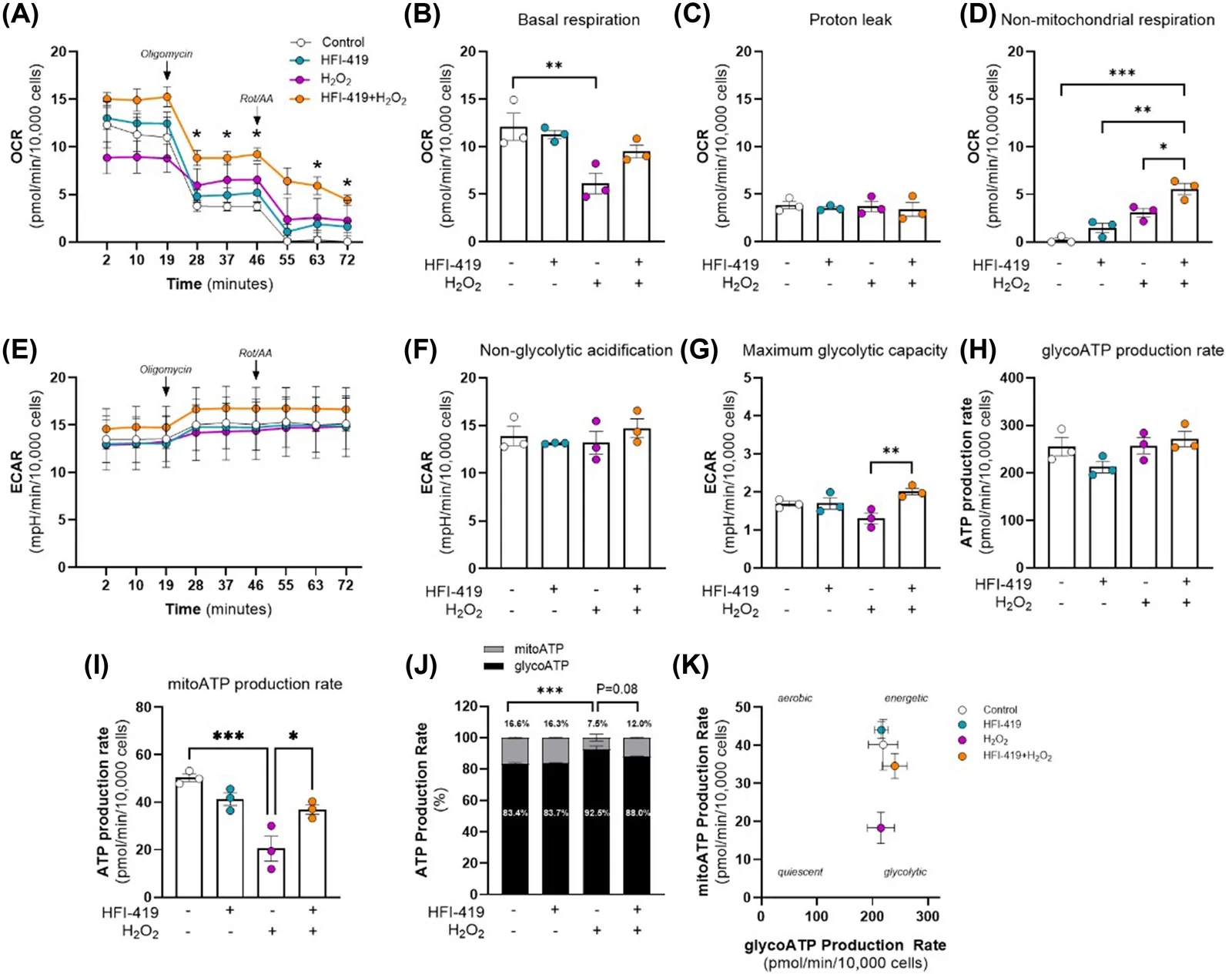
HFI-419 prevents H_2_O_2_-induced oxidative stress in HK-2 cells Metabolic profiling and ATP production rate of HK-2 cells treated with H_2_O_2_ (1.3 M) ± HFI-419 (0.01 μM) for 4 h. OCR presented as (**A**) the kinetic profile, (**B**) mean basal respiration, (**C**) mean proton leak, and (**D**) mean non-mitochondrial respiration. ECAR presented as (**E**) a kinetic profile, (**F**) mean non-glycolytic acidification, and (**G**) maximum glycolytic capacity. (**H**) Mean glycoATP production rates, (**I**) mean mitoATP production rates, (**J**) relative proportion of ATP production attributed to mitoATP and glycoATP, and (**K**) energetic map indicating shifts in metabolic phenotypes after treatment. Data are mean ± SEM; *n =* 3 from independent experiments, each performed with three technical replicates. (A,E,J,K) Data analysed via two-way ANOVA followed by Sidak’s multiple comparisons test. For panels (A,E), treatment groups were compared within each timepoint. For panels (J,K), *P*-values represent the main effects of H_2_O_2_ and HFI-419; *P<0.05, ***P<0.001. (B–D,F–I) Data analysed via one-way ANOVA with Sidak’s multiple comparisons tests. Pairwise comparisons were control versus H_2_O_2_, control versus HFI-419, H_2_O_2_ versus HFI-419 and control versus HFI-419+ H_2_O_2_; *P<0.05, ***P<0.001. Rot/AA, rotenone/antimycin A.

**Figure 6:**
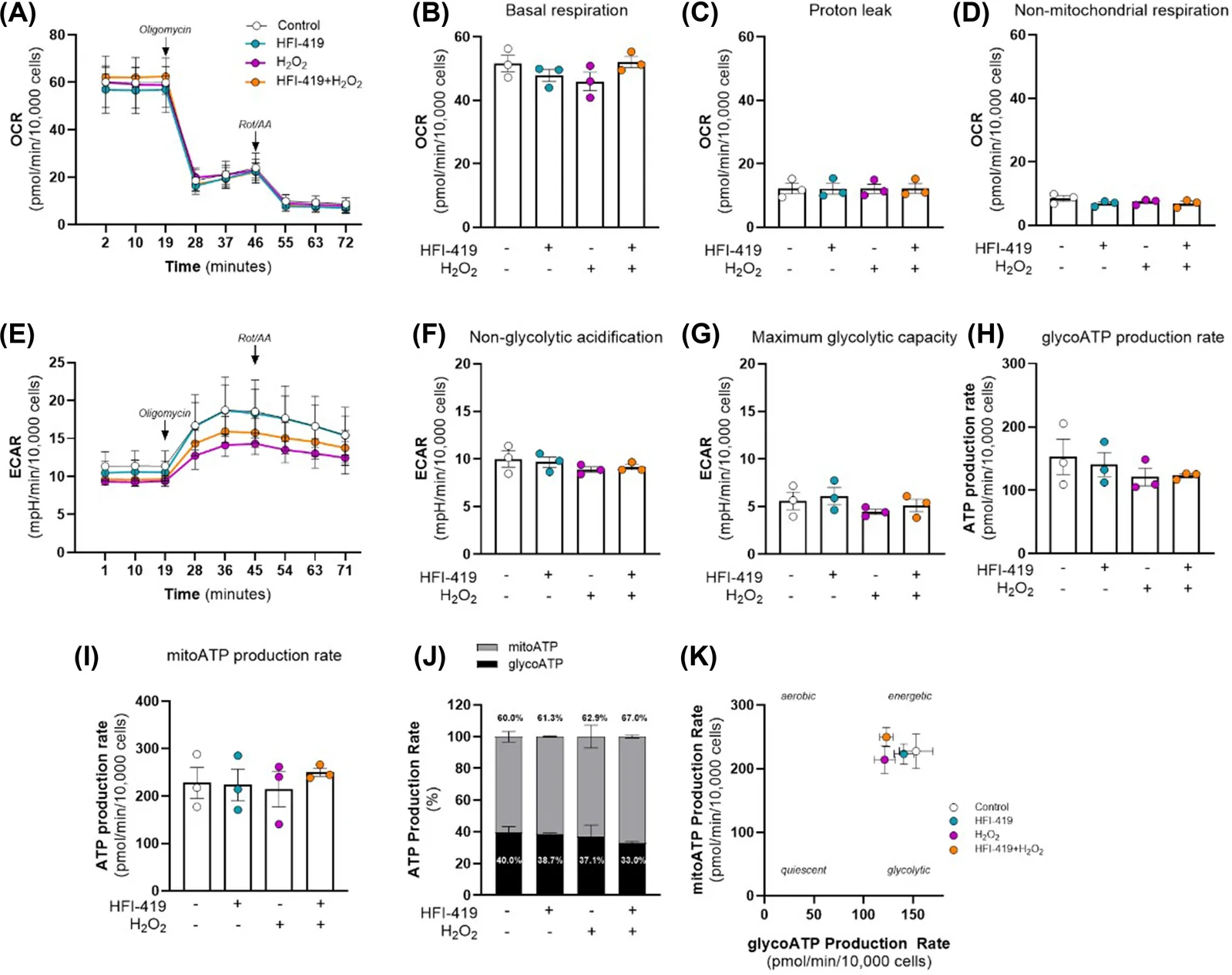
IRAP inhibition has no effect on the metabolic profile of MDCK cells Metabolic profiling and ATP production rate of MDCK cells treated with H_2_O_2_ (1.3 M) ± HFI-419 (0.01 μM) for 4 h. OCR presented as (**A**) the kinetic profile, (**B**) mean basal respiration, (**C**) mean proton leak, and (**D**) mean non-mitochondrial respiration. ECAR presented as (**E**) a kinetic profile, (**F**) mean non-glycolytic acidification, and (**G**) maximum glycolytic capacity. (**H**) Mean glycoATP production rates, (**I**) mean mitoATP production rates, (**J**) relative proportion of ATP production attributed to mitoATP and glycoATP, and (**K**) energetic map indicating shifts in metabolic phenotypes after treatment. Data are mean ± SEM; *n =* 3 from independent experiments, each performed with three technical replicates. (A,E,J,K) Data analysed via two-way ANOVA followed by Sidak’s multiple comparisons test; For panels (A,E), treatment groups were compared within each timepoint, and for panels (J,K), no significant main effects of H_2_O_2_ and HFI-419 were detected. (B–D,F–I). Data analysed via one-way ANOVA, with no significant effects detected. Rot/AA, rotenone/antimycin A.`

Given that mitochondrial dysfunction plays a central role in renal tubular senescence, we used a Seahorse XF ATP rate assay to determine whether the anti-senescence properties of HFI-419 were associated with shifts in mitochondrial ATP production. HK-2 cells were treated with or without HFI-419 under control or oxidative stress conditions for 4 h. The kinetic OCR and ECAR profiles of treated HK-2 cells are presented in Figure 5A,E. Basal respiration was suppressed by ∼50% following H_2_O_2_ treatment but enhanced upon co-treatment with HFI-419 (*P<*0.01 versus control, Figure 5B). Inhibition of ATP synthesis at complex V of the electron transport chain by oligomycin did not affect the OCR in terms of proton leak in any of the treatment groups (Figure 5C). Inhibition of complexes I and III of the electron transport chain by Rot/AA (indicative of non-mitochondrial respiration) was associated with enhanced OCR in cells treated with H_2_O_2_ and HFI-419 (*P<*0.05 versus H_2_O_2_, Figure 5D). Non-glycolytic acidification was similar across the treatment groups (Figure 6F). The maximum glycolytic capacity was enhanced by treatment with H_2_O_2_ + HFI-419, compared with H_2_O_2_ stress alone (*P<*0.01, Figure 5G). Under standard culture conditions, HK-2 cells primarily generated ATP via glycolysis (Figure 5J). Upon H_2_O_2_ treatment, there was a 60% reduction in mitochondrial ATP production (*P<*0.001 versus control, Figure 5I,K) with cells shifting towards a more glycolytic phenotype compared with control (Figure 5J,K). This metabolic shift was partially attenuated by IRAP inhibition, which enhanced the mitoATP production rate (*P<*0.05 versus H_2_O_2_, Figure 5I). Together, these findings demonstrate that, in a pro-senescence environment, IRAP inhibition can modulate proximal tubule mitochondrial respiration by enhancing mitochondrial capacity and shifting cells from a glycolytic to a more energetically favourable metabolic phenotype.

### MDCK cells are resistant to H_2_O_2_-induced mitochondrial dysfunction

Basal MDCK OCR was unaffected by 4 h of H_2_O_2_ or HFI-419 treatments when compared with control (Figure 6A,B). Inhibition of ATP synthesis at complex V by oligomycin did not affect proton leak between groups, nor did inhibition of complex I and III of the electron transport chain by Rot/AA have an effect (Figure 6A). Proton leak and non-mitochondrial respiration were equivalent between groups (Figure 6C,D). Furthermore, non-glycmplex I and Iolytic acidification and maximum glycolytic capacity derived from the ECAR kinetic profile were similar between all groups (Figure 6E–G). MDCK cells showed an energetic phenotype, producing more mitoATP than glycoATP (Figure 6J,K). Neither H_2_O_2_ nor HFI-419 treatments influenced the metabolic phenotype of MDCK cells (Figure 6H–K). This shows that IRAP inhibition does not affect mitochondrial ATP production in MDCK cells, unlike HK-2 cells, and points towards a differential role of IRAP between the renal tubule segments.

## Discussion

This is the first report demonstrating a role for IRAP in the age-associated decline in renal function and pathology. We show that global IRAP deficiency preserved renal function and conferred marked anti-senescent and anti-fibrotic effects in aged male and female mice. Second, pharmacological IRAP inhibition over a four-week window was sufficient to blunt GFR decline and suppress cellular senescence markers in elderly WT mice. Finally, we demonstrated that the anti-senescent effects of IRAP inhibition are associated with shifts in metabolic phenotype in cultured proximal tubule cells. Taken together, these data support our hypothesis that IRAP drives renal injury under conditions of stress and represents a novel therapeutic target for kidney ageing.

Ageing is a complex process that affects multiple organ systems and cell types asynchronously, with the kidney particularly vulnerable [33,34]. Yet, most kidney disease models involve aggressive and accelerated disease in young and healthy male mice. For this reason, we tested the effects of IRAP deficiency in naturally aged mice. With the median lifespan of C57BL/6J mice reported to be 20–32 months [35], our animal cohorts of 15–28 months of age represent middle-aged through elderly populations. The progressive decline in kidney function in aged WT mice coincided with glomerulosclerosis, albuminuria, renal fibrosis and senescence, mirroring the ageing human kidney [34]. Marked attenuation of several functional and structural ageing signs in IRAP-deficient mice indicates a novel role of the IRAP axis in renal ageing. Importantly, the protective effects of IRAP deficiency were independent of arterial pressure in male mice. This finding is consistent with our prior report showing that IRAP deficiency does not affect arterial pressure in female mice [14]. Pharmacologic inhibition of IRAP with HFI-419 produced similar GFR-sparing and anti-fibrotic effects in aged WT mice, suggesting that IRAP deficiency exerts protective effects at the molecular and cellular levels.

Chronic senescent burden has been shown to play a propagating role in renal fibrosis and ageing in mammals [15,17]. The near-absence of SA-β-gal activity and attenuation of p21 nuclear expression in aged IRAP KO mice, in striking contrast to widespread SA-β-gal activity and p21 expression in aged WT mice, suggests that IRAP may drive renal senescence and subsequent renal injury. Suppression of the p16^INK4a^/p21 cell cycle arrest pathways observed with global IRAP deficiency and HFI-419 treatment confirmed cell cycle arrest at the transcriptional level. To the best of our knowledge, this is the first study to show that diminution of IRAP exerts anti-senescent effects. Evidence indicates that activation of p21 occurs in the early stages of ageing, primarily triggered via DNA damage by reactive oxygen species [15,36], whereas p16^INK4a^ activation is more reflective of persistent exposure to cellular stress [31]. For example, p16^INK4a^ expression correlates with age and degenerative morphological changes in human renal cortex samples, including glomerulosclerosis and interstitial fibrosis [37–39]. p16^INK4a^ expression was detected in all components of the human nephron, but particularly in tubular cells, of aged kidneys [38], similar to the predominance of SA-β-gal activity in proximal tubules in our aged WT mice. Dampening of these pro-senescent pathways through IRAP deficiency supports the idea that IRAP inhibition may act as a novel senotherapeutic and anti-fibrotic agent. As cellular senescence is implicated in age-related decline in healthspan and lifespan, it is important for future studies to determine whether the renoprotective effects of IRAP deficiency translate into improved survival.

Disruption of renal energy metabolism is a key pro-senescent and pro-fibrotic stimulus [15,40]. IRAP inhibition conferred resistance to a senescent cell fate outcome in proximal tubule cells cultured in an oxidative stress milieu. This appears to be achieved through biogenetic mechanisms, with IRAP inhibition preventing the oxidative stress-induced shifts in cellular phenotype towards glycolysis. The metabolic function of the slowly proliferative MDCK collecting duct cell line was unaffected by oxidative stress or IRAP inhibition. This is consistent with the greater literature showing tubule-specific metabolism [41–44]. Proximal tubule cells have unique energy demands, with more active transport mechanisms than any other renal cell type to accommodate the reabsorption of ∼70% of the filtrate that passes through the glomerulus [40,42]. Proximal tubule cells are packed with mitochondria to meet these high energy requirements and preferentially metabolise fatty acids such as palmitate via β-oxidation to produce a large amount of fatty acid-derived ATP [42,43,45]. For this reason, combined with limited capacity for glycolysis [45], the proximal tubule is vulnerable to renal stress. A metabolic switch towards inefficient glycolytic pathways has been reported in rodent models of primary kidney injury and chronic kidney disease [45], as well as in several *in vivo* models of renal injury, including ischemia– reperfusion injury [43] and streptozotocin-induced diabetic kidney disease [46] in mice. TGF-β and HIF-1α signalling have been shown to drive this glycolytic switch in human proximal tubule *in vitro* models of senescence, either through late passaging or exposure to hypoxia [41,44]. The ability of IRAP antagonism to reverse oxidative stress-induced glycolytic shifts may underpin its anti-senescent actions in the proximal tubule. We explored whether the anti-senescent actions of IRAP affect wound healing, given that accumulating senescent cells create a non-resolving, inflammatory niche that impairs cell migration and re-epithelialisation in aged and diabetic skin [47]. IRAP inhibition abrogated dysregulated wound healing under oxidative stress conditions, confirming our observations that wound healing is not impaired in IRAP deficient mice following surgical procedures in both our present and prior study [14]. IRAP inhibition may, therefore, block inflammation and enable epithelial repair.

Despite IRAP being highly expressed in the kidney, current evidence for its function is derived from a small number of associational studies, which point to a role in kidney injury. The IRAP gene, *Lnpep*, was identified in a transcriptome-wide association study of individuals of European ancestry as a druggable target associated with kidney disease [48]. In support of this, elevated IRAP expression was reported in Wistar rats subjected to cisplatin-induced [49] and breast cancer-induced kidney injury [50]. Reno-protective effects were previously reported in global IRAP-deficient male mice treated with lipopolysaccharide, where glomerular fibrin deposition was attenuated compared with wild types [51]. Although the functional significance was not reported, the protective effects of IRAP deficiency on the glomerulus are consistent with our study findings. Moreover, quercetin, a flavonoid with antioxidant and anti-inflammatory properties that enhance mitochondrial function [52,53], down-regulates *Lnpep* in human mesenchymal stem cells [54] and HepG2 cells [55]. Taken together, this indirect evidence supports our conclusion that IRAP inhibition has anti-senescent properties.

IRAP, as a metalloprotease, has been reported to have additional, diverse biological functions. IRAP mediates substrate catalysis via the C-terminal domain, of which vasopressin is the best characterised substrate and contributes to fluid homeostasis [14]. Trafficking motifs in the IRAP N-terminal domain regulate intracellular vesicular trafficking in muscle, adipose, immune cells and the brain, particularly the mobilization of GLUT-4 vesicles in response to insulin [8]. The well-documented cognitive-enhancing effects of IRAP inhibitor treatment appear to occur in part via GLUT4-mediated enhancement of neuronal glucose uptake and neuronal remodelling. However, the role of IRAP in the kidney appears to be independent of facilitation of glucose uptake, as IRAP and GLUT-4 are not colocalised within the renal tubules in the kidney [9]. The IRAP inhibitor used in the present study, HFI-419, belongs to a 2-amino-4H-benzopyran group that binds specifically to the catalytic domain of IRAP [56]. In our study, HFI-419 attenuated expression of senescence-associated transcripts and p21-positive cell burden while preserving GFR and reducing renal collagen accumulation. However, HFI-419 did not improve all measures of renal ageing, including albuminuria and glomerulosclerosis. As HFI-419 inhibits IRAP enzymatic activity without altering IRAP protein abundance, these differences may indicate that complete loss of IRAP has broader biological effects than inhibition of its catalytic activity alone. Further, we cannot exclude the possibility that the protective glomerular effects of IRAP deficiency are secondary to systemic or tubulointerstitial changes, rather than the direct effect of IRAP deficiency within glomerular cells. Future studies are required to distinguish the relative contributions of IRAP’s C-terminal and N-terminal domains to kidney function and ageing, and to examine the contributions of IRAP to glomerular pathology specifically.

We acknowledge several limitations and future directions of the present study. Although immunohistochemistry demonstrated an age-associated increase in renal IRAP expression, whether ageing is associated with enhanced IRAP catalytic activity and/or altered signalling mediated by its N-terminal trafficking domain remains to be determined. Future studies may help define how IRAP contributes to age-related kidney dysfunction and whether specific IRAP pathways represent therapeutic ageing targets. Oxidative stress, examined *in vitro*, does not encompass the full spectrum of senescent stimuli in ageing. Whether the protective actions of IRAP deficiency are underpinned by anti-senescent effects or are a consequence of unresolved signalling pathways remains to be determined. Recent clinical studies indicate that intermittent senolytic treatments, such as the combination of dasatinib and quercetin [23], may quell senescent burden for several weeks before treatment is required again [15,23]. Notably, quercetin down-regulates IRAP transcriptionally *in vitro* [54,55]. Given that intermittent treatment aims to reduce symptoms and increase drug compliance, future studies may consider whether intermittent IRAP inhibition promotes senoprotective effects. Our findings support further research to delineate IRAP’s mechanism of action within the kidney, to determine whether IRAP deficiency produces protective effects in kidney diseases with overlapping pathological features of ageing, and whether benefits of IRAP deficiency extend to the broader cardiovascular system.

In conclusion, our study addresses a fundamental knowledge gap regarding the actions of IRAP in the kidney. We show that IRAP deficiency in both male and female mice is protective against hallmarks of kidney ageing, including kidney function decline, senescence and fibrosis. Through *in vitro* studies, we propose a putative role for IRAP in driving the metabolic switch towards glycolysis and promoting a chronic senescent cell fate in proximal tubules under conditions of oxidative stress. Strategies to inhibit IRAP may constitute an important advance for patients with kidney disease driven by cellular senescence.

## Clinical perspectives

- Age-related renal decline is a major contributor to cardiovascular morbidity, yet there are no established therapies to target the biological processes that drive renal ageing.
- Our studies identify IRAP as a mediator of renal ageing, with IRAP deficiency conferring senoprotective and anti-fibrotic effects in aged mice. Further, pharmacologic inhibition of IRAP *in vitro* enhanced proximal tubule metabolic function under pro-senescent stress.
- Targeting IRAP signalling represents a promising senoprotective strategy for kidney ageing and cardiovascular sequelae.

## Supplementary Material

Supplementary Figures S1-S4 and Table S1

## Data Availability

Data and protocols will be made available upon reasonable request.

## Open Access

Open access for this article was enabled by the participation of Monash University in an all-inclusive Read & Publish agreement with Portland Press and the Biochemical Society under an agreement with CAUL.

## Abbreviations

Ang: angiotensin
BP: blood pressure
ECAR: extracellular acidification rate
FITC: fluorescein isothiocyanate
GFR: glomerular filtration rate
GSI: glomerulosclerosis index
IRAP: insulin-regulated aminopeptidase
KO: knockout
MDCK: Madin–Darby canine kidney
NIC: non-invasive clearance
PAS: periodic acid-Schiff
RAS: renin-angiotensin system
SA-β-gal: senescence-associated beta-galactosidase
SEM: standard error of mean
WT: wild-type

## References

[1] Colafella K.M.M. and Denton K.M. (2018) Sex-specific differences in hypertension and associated cardiovascular disease. Nat. Rev. Nephrol. 14, 185–201 29380817 10.1038/nrneph.2017.189

[2] de Cavanagh E.M.V., Inserra F. and Ferder L. (2015) Angiotensin II blockade: how its molecular targets may signal to mitochondria and slow aging. Coincidences with calorie restriction and mTOR inhibition. Am. J. Physiol. Heart Circ. Physiol. 309, H15–H44 25934099 10.1152/ajpheart.00459.2014

[3] Park B.M., Cha S.A., Lee S.H. and Kim S.H. (2016) Angiotensin IV protects cardiac reperfusion injury by inhibiting apoptosis and inflammation via AT4R in rats. Peptides 79, 66–74 27038740 10.1016/j.peptides.2016.03.017

[4] Bai W.-w., Wang H., Gao C.-h., Liu K.-y., Guo B.-x., Jiang F. et al. (2021) Continuous infusion of Angiotensin IV protects against acute myocardial infarction via the inhibition of inflammation and autophagy. Oxidative Med. Cell. Longevity 2021, 2860488 34950416 10.1155/2021/2860488 PMC8691990

[5] Handa R.K., Krebs L.T., Harding J.W. and Handa S.E. (1998) Angiotensin IV AT4-receptor system in the rat kidney. Am. J. Physiol. Renal Physiol. 274, F290–F299 9486224 10.1152/ajprenal.1998.274.2.F290

[6] Lew R.A., Mustafa T., Ye S., McDowall S.G., Chai S.Y. and Albiston A.L. (2003) Angiotensin AT4 ligands are potent, competitive inhibitors of insulin regulated aminopeptidase (IRAP). J. Neurochem. 86, 344–350 12871575 10.1046/j.1471-4159.2003.01852.x

[7] Matsumoto H., Nagasaka T., Hattori A., Rogi T., Tsuruoka N., Mizutani S. et al. (2001) Expression of placental leucine aminopeptidase/oxytocinase in neuronal cells and its action on neuronal peptides. Eur. J. Biochem. 268, 3259–3266 11389728 10.1046/j.1432-1327.2001.02221.x

[8] Vear A., Gaspari T., Thompson P. and Chai S.Y. (2020) Is there an interplay between the functional domains of IRAP? Front. Cell Dev. Biol. 8, 585237 33134302 10.3389/fcell.2020.585237 PMC7550531

[9] Albiston A.L., Yeatman H.R., Pham V., Fuller S.J., Diwakarla S., Fernando R.N. et al. (2011) Distinct distribution of GLUT4 and insulin regulated aminopeptidase in the mouse kidney. Regul. Pept. 166, 83–89 20851149 10.1016/j.regpep.2010.09.003

[10] Prieto I., Villarejo A.B., Segarra A.B., Wangensteen R., Banegas I., de Gasparo M. et al. (2015) Tissue distribution of CysAP activity and its relationship to blood pressure and water balance. Life Sci. 134, 73–78 26006037 10.1016/j.lfs.2015.04.023

[11] Prieto I., Segarra A.B., de Gasparo M., Martínez-Cañamero M. and Ramírez-Sánchez M. (2018) Divergent profile between hypothalamic and plasmatic aminopeptidase activities in WKY and SHR. Influence of beta-adrenergic blockade. Life Sci. 192, 9–17 29155297 10.1016/j.lfs.2017.11.022

[12] Villarejo A., Segarra A., Ramírez M., Banegas I., Wangensteen R., de Gasparo M. et al. (2012) Angiotensinase and vasopressinase activities in hypothalamus, plasma, and kidney after inhibition of angiotensin-converting enzyme: basis for a new working hypothesis. Horm. Metab. Res. 44, 152–154 22203440 10.1055/s-0031-1299693

[13] Zuchowski Y., Carty J., Terker A.S., Bock F., Trapani J.B., Bhave G. et al. (2023) Insulin-regulated aminopeptidase is required for water excretion in response to acute hypotonic stress. Am. J. Physiol.-Renal Physiol. 324, F521–F531 36995926 10.1152/ajprenal.00318.2022 PMC10202483

[14] Walton S.L., Mirabito Colafella K.M., Ansari A., Chai S.Y. and Denton K.M. (2020) Insulin-regulated aminopeptidase deficiency impairs cardiovascular adaptations and placental development during pregnancy. Clin. Sci. 134, 3213–3228 33252660 10.1042/CS20201233 PMC7733041

[15] Huang W., Hickson L.J., Eirin A., Kirkland J.L. and Lerman L.O. (2022) Cellular senescence: the good, the bad and the unknown. Nat. Rev. Nephrol. 18, 611–627 35922662 10.1038/s41581-022-00601-z PMC9362342

[16] Sturmlechner I., Durik M., Sieben C.J., Baker D.J. and Van Deursen J.M. (2017) Cellular senescence in renal ageing and disease. Nat. Rev. Nephrol. 13, 77 28029153 10.1038/nrneph.2016.183

[17] Knoppert S.N., Valentijn F.A., Nguyen T.Q., Goldschmeding R. and Falke L.L. (2019) Cellular senescence and the kidney: potential therapeutic targets and tools. Front. Pharmacol. 10, 770 31354486 10.3389/fphar.2019.00770 PMC6639430

[18] Hickson L.J., Langhi Prata L.G.P., Bobart S.A., Evans T.K., Giorgadze N., Hashmi S.K. et al. (2019) Senolytics decrease senescent cells in humans: preliminary report from a clinical trial of Dasatinib plus Quercetin in individuals with diabetic kidney disease. eBioMedicine 47, 446–456 31542391 10.1016/j.ebiom.2019.08.069 PMC6796530

[19] Schmitt R. and Melk A. (2017) Molecular mechanisms of renal aging. Kidney Int. 92, 569–579 28729036 10.1016/j.kint.2017.02.036

[20] Baker D.J., Wijshake T., Tchkonia T., LeBrasseur N.K., Childs B.G., Van De Sluis B. et al. (2011) Clearance of p16 Ink4a-positive senescent cells delays ageing-associated disorders. Nature 479, 232–236 22048312 10.1038/nature10600 PMC3468323

[21] Xu M., Pirtskhalava T., Farr J.N., Weigand B.M., Palmer A.K., Weivoda M.M. et al. (2018) Senolytics improve physical function and increase lifespan in old age. Nat. Med. 24, 1246–1256 29988130 10.1038/s41591-018-0092-9 PMC6082705

[22] Xu M., Bradley E.W., Weivoda M.M., Hwang S.M., Pirtskhalava T., Decklever T. et al. (2017) Transplanted senescent cells induce an osteoarthritis-like condition in mice. J. Gerontol.: Series A. 72, 780–785 27516624 10.1093/gerona/glw154 PMC5861939

[23] Hickson L.J., Prata L.G.L., Bobart S.A., Evans T.K., Giorgadze N., Hashmi S.K. et al. (2019) Senolytics decrease senescent cells in humans: preliminary report from a clinical trial of Dasatinib plus Quercetin in individuals with diabetic kidney disease. eBioMedicine 47, 446–456 31542391 10.1016/j.ebiom.2019.08.069 PMC6796530

[24] Albiston A.L., Fernando R.N., Yeatman H.R., Burns P., Ng L., Daswani D. et al. (2010) Gene knockout of insulin-regulated aminopeptidase: loss of the specific binding site for angiotensin IV and age-related deficit in spatial memory. Neurobiol. Learn. Mem. 93, 19–30 19660563 10.1016/j.nlm.2009.07.011

[25] Brown R.D., Hilliard L.M., Head G.A., Jones E.S., Widdop R.E. and Denton K.M. (2012) Sex differences in the pressor and tubuloglomerular feedback response to angiotensin II. Hypertension 59, 129–135 22124434 10.1161/HYPERTENSIONAHA.111.178715

[26] Friedemann J., Heinrich R., Shulhevich Y., Raedle M., William-Olsson L., Pill J. et al. (2016) Improved kinetic model for the transcutaneous measurement of glomerular filtration rate in experimental animals. Kidney Int. 90, 1377–1385 27665115 10.1016/j.kint.2016.07.024

[27] Gallo L.A., Walton S.L., Mazzuca M.Q., Tare M., Parkington H.C., Wlodek M.E. et al. (2018) Uteroplacental insufficiency temporally exacerbates salt‐induced hypertension associated with a reduced natriuretic response in male rat offspring. J. Physiol. 596, 5859–5872 29604087 10.1113/JP275655 PMC6265551

[28] Walton S.L., Mazzuca M.Q., Tare M., Parkington H.C., Wlodek M.E., Moritz K.M. et al. (2018) Angiotensin receptor blockade in juvenile male rat offspring: Implications for long-term cardio-renal health. Pharmacol. Res. 134, 320–331 29870806 10.1016/j.phrs.2018.06.001

[29] Wang Y., Han L., Shen M., Jones E.S., Spizzo I., Walton S.L. et al. (2020) Serelaxin and the AT2 receptor agonist CGP42112 evoked a similar, nonadditive, cardiac antifibrotic effect in high salt-fed mice that were refractory to candesartan cilexetil. ACS Pharmacol. Transl. Sci. 3, 76–87 32259090 10.1021/acsptsci.9b00095 PMC7088889

[30] Dimri G.P., Lee X., Basile G., Acosta M., Scott G., Roskelley C. et al. (1995) A biomarker that identifies senescent human cells in culture and in aging skin *in vivo*. Proc. Natl. Acad. Sci. U.S.A. 92, 9363–9367 7568133 10.1073/pnas.92.20.9363 PMC40985

[31] Ansari A., Walton S.L. and Denton K.M. (2023) Sex-and age-related differences in renal and cardiac injury and senescence in stroke-prone spontaneously hypertensive rats. Biol. Sex Diff. 14, 33 37217968 10.1186/s13293-023-00519-6 PMC10201739

[32] Walton S.L., Tjongue M., Tare M., Kwok E., Probyn M., Parkington H.C. et al. (2019) Chronic low alcohol intake during pregnancy programs sex-specific cardiovascular deficits in rats. Biol. Sex Differ. 10, 21 31010438 10.1186/s13293-019-0235-9 PMC6477739

[33] Bolignano D., Mattace-Raso F., Sijbrands E.J. and Zoccali C. (2014) The aging kidney revisited: a systematic review. Ageing Res. Rev. 14, 65–80 24548926 10.1016/j.arr.2014.02.003

[34] Denic A., Glassock R.J. and Rule A.D. (2016) Structural and functional changes with the aging kidney. Adv. Chronic Kidney Dis. 23, 19–28 26709059 10.1053/j.ackd.2015.08.004 PMC4693148

[35] Spiridonova O., Kriukov D., Nemirovich-Danchenko N. and Peshkin L. (2024) On standardization of controls in lifespan studies. Aging (Albany NY) 16, 3047–3055 38421245 10.18632/aging.205604 PMC10929834

[36] Hsu C.-H., Altschuler S.J. and Wu L.F. (2019) Patterns of early p21 dynamics determine proliferation-senescence cell fate after chemotherapy. Cell 178, 361.e312–373.e312 10.1016/j.cell.2019.05.041 PMC6688629 31204100

[37] Idda M.L., McClusky W.G., Lodde V., Munk R., Abdelmohsen K., Rossi M. et al. (2020) Survey of senescent cell markers with age in human tissues. Aging (Albany NY) 12, 4052 32160592 10.18632/aging.102903 PMC7093180

[38] Melk A., Schmidt B.M.W., Takeuchi O., Sawitzki B., Rayner D.C. and Halloran P.F. (2004) Expression of p16INK4a and other cell cycle regulator and senescence associated genes in aging human kidney. Kidney Int. 65, 510–520 14717921 10.1111/j.1523-1755.2004.00438.x

[39] Chkhotua A.B., Gabusi E., Altimari A., D’Errico A., Yakubovich M., Vienken J. et al. (2003) Increased expression of p16(INK4a) and p27(Kip1) cyclin-dependent kinase inhibitor genes in aging human kidney and chronic allograft nephropathy. Am. J. Kidney Dis. 41, 1303–1313 12776284 10.1016/S0272-6386(03)00363-9

[40] Doke T. and Susztak K. (2022) The multifaceted role of kidney tubule mitochondrial dysfunction in kidney disease development. Trends Cell Biol. 32, 841–853 35473814 10.1016/j.tcb.2022.03.012 PMC9464682

[41] Dickman K.G. and Mandel L.J. (1990) Differential effects of respiratory inhibitors on glycolysis in proximal tubules. Am. J. Physiol. 258, F1608–F1615 2163215 10.1152/ajprenal.1990.258.6.F1608

[42] Hall A.M., Unwin R.J., Parker N. and Duchen M.R. (2009) Multiphoton imaging reveals differences in mitochondrial function between nephron segments. J. Am. Soc. Nephrol. 20, 1293–1302 19470684 10.1681/ASN.2008070759 PMC2689904

[43] Lan R., Geng H., Singha P.K., Saikumar P., Bottinger E.P., Weinberg J.M. et al. (2016) Mitochondrial pathology and glycolytic shift during proximal tubule atrophy after ischemic AKI. J. Am. Soc. Nephrol. 27, 3356–3367 27000065 10.1681/ASN.2015020177 PMC5084876

[44] Lu Y., Wang J., Dapeng C., Wu D., Cai G. and Chen X. (2016) Bioinformatics analysis of proteomics profiles in senescent human primary proximal tubule epithelial cells. BMC Nephrol. 17, 39 27036204 10.1186/s12882-016-0249-z PMC4818421

[45] Schaub J.A., Venkatachalam M.A. and Weinberg J.M. (2021) Proximal tubular oxidative metabolism in acute kidney injury and the transition to CKD. Kidney360 2, 355–364 35373028 10.34067/KID.0004772020 PMC8740982

[46] Wu M., Li S., Yu X., Chen W., Ma H., Shao C. et al. (2019) Mitochondrial activity contributes to impaired renal metabolic homeostasis and renal pathology in STZ-induced diabetic mice. Am. J. Physiol.-Renal Physiol. 317, F593–F605 31268353 10.1152/ajprenal.00076.2019

[47] Kita A., Yamamoto S., Saito Y. and Chikenji T.S. (2024) Cellular senescence and wound healing in aged and diabetic skin. Front. Physiol. 15, 1344116 38440347 10.3389/fphys.2024.1344116 PMC10909996

[48] Lu Y.-Q. and Wang Y. (2024) Multi-omic analysis reveals genetic determinants and therapeutic targets of chronic kidney disease and kidney function. Int. J. Mol. Sci. 25, 6033 38892221 10.3390/ijms25116033 PMC11172763

[49] Quesada A., Vargas F., Montoro-Molina S., O’Valle F., Rodríguez-Martínez M.D., Osuna A. et al. (2012) Urinary aminopeptidase activities as early and predictive biomarkers of renal dysfunction in cisplatin-treated rats. PloS One 7, e40402 22792302 10.1371/journal.pone.0040402 PMC3390365

[50] Martínez-Martos J.M., Carrera-González M., Sánchez-Agesta R., García M.J. and Ramírez-Expósito M.J. (2018) Kidney aminopeptidase activities are related to renal damage in experimental breast cancer. J. Clin. Mol. Med. 1, 1–8 10.15761/JCMM.1000105

[51] Numaguchi Y., Ishii M., Kubota R., Morita Y., Yamamoto K., Matsushita T. et al. (2009) Ablation of Angiotensin IV receptor attenuates hypofibrinolysis via PAI-1 downregulation and reduces occlusive arterial thrombosis. Arterioscler. Thromb. Vasc. Biol. 29, 2102–2108 19745198 10.1161/ATVBAHA.109.195057

[52] Carrillo-Garmendia A., Madrigal-Perez L.A. and Regalado-Gonzalez C. (2024) The multifaceted role of quercetin derived from its mitochondrial mechanism. Mol. Cell. Biochem. 479, 1985–1997 37656383 10.1007/s11010-023-04833-w

[53] Li X., Wang H., Gao Y., Li L., Tang C., Wen G. et al. (2016) Protective effects of quercetin on mitochondrial biogenesis in experimental traumatic brain injury via the Nrf2 signaling pathway. PloS One 11, e0164237 27780244 10.1371/journal.pone.0164237 PMC5079551

[54] Geng L., Liu Z., Zhang W., Li W., Wu Z., Wang W. et al. (2019) Chemical screen identifies a geroprotective role of quercetin in premature aging. Protein Cell. 10, 417–435 30069858 10.1007/s13238-018-0567-y PMC6538594

[55] Magkoufopoulou C., Claessen S.M.H., Jennen D.G.J., Kleinjans J.C.S. and van Delft J.H.M. (2011) Comparison of phenotypic and transcriptomic effects of false-positive genotoxins, true genotoxins and non-genotoxins using HepG2 cells. Mutagenesis 26, 593–604 21632981 10.1093/mutage/ger021

[56] Albiston A.L., Morton C.J., Ng H.L., Pham V., Yeatman H.R., Ye S. et al. (2008) Identification and characterization of a new cognitive enhancer based on inhibition of insulin‐regulated aminopeptidase. FASEB J. 22, 4209–4217 18716029 10.1096/fj.08-112227

