# Supplementary material for: IRAP deficiency preserves renal function and attenuates pathology in aged mice
Source: Clin Sci (Lond). 2026 Sep 16;140(10):2035–51. doi: 10.1042/CS20261576 (PMC13591257; doi:10.1042/CS20261576)
Supplement: Supplementary file 1 [file CS-2026-1576_supp.pdf]

Supplementary materials

IRAP deficiency preserves renal function and attenuates pathology in aged mice

Sarah L Walton[1,2]*, Aneesa Ansari[1,2]*, Katrina M Mirabito Colafella[1,2], Lucinda M Hilliard Krause[1,2], Giannie Barsha[1,2], Michelle M Kett[1,6], Siew Yeen Chai[1,3], Vesna D Garovic[4,5], Kate M Denton[1,2]

*Authors contributed equally

Table of Contents

Supplemental Table 1. Accession codes for TaqMan™ Gene Expression Assays

Supplemental Figure 1. Body and kidney weights of aged mice.

Supplemental Figure 2. IRAP deficiency prevents age-related decline in renal function and accumulation of senescent cells in female mice.

Supplemental Figure 3. IRAP is expressed in the mouse kidney and is upregulated in ageing.

Supplemental Figure 4. IRAP is expressed in HK-2 and MDCK cell lines.

Supplemental Data

Table S1. Accession codes for TaqMan^{TM} Gene Expression Assays

Gene	Accession code	Supplier
Cdkn2a	Mm00494449_m1	Thermo Fisher
Cdkn1a	Mm00432448_m1	Thermo Fisher
Trp53	Mm_01731290_g1	Thermo Fisher
18S	X03205.1	Applied Biosystems

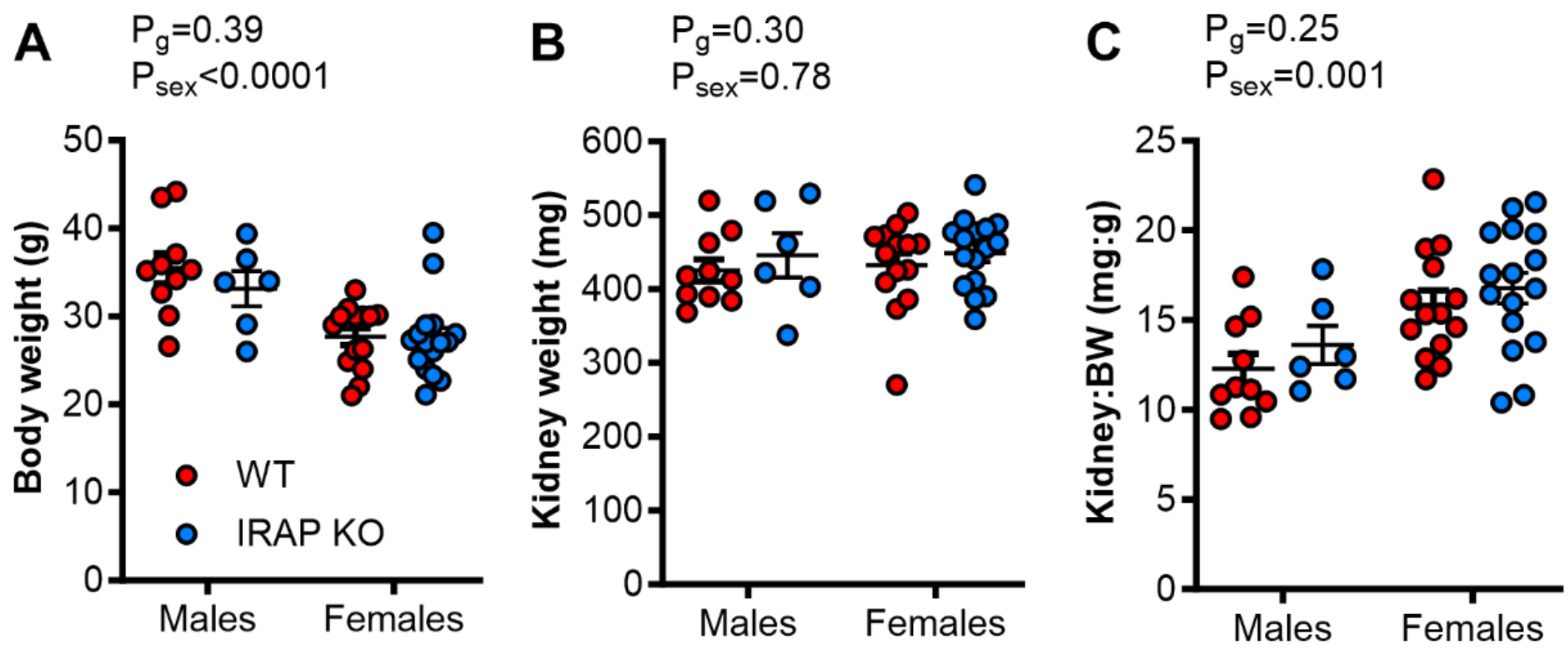

Figure S1. Body and kidney weights of aged mice.

(A) Body weight, (B) total kidney weight and (C) total kidney weight relative to body weight in male and female WT (open bars) and IRAP KO (closed bars) mice at 23 months of age. N=6-16. Data are analysed via two-way ANOVA; no significant interactions were found.

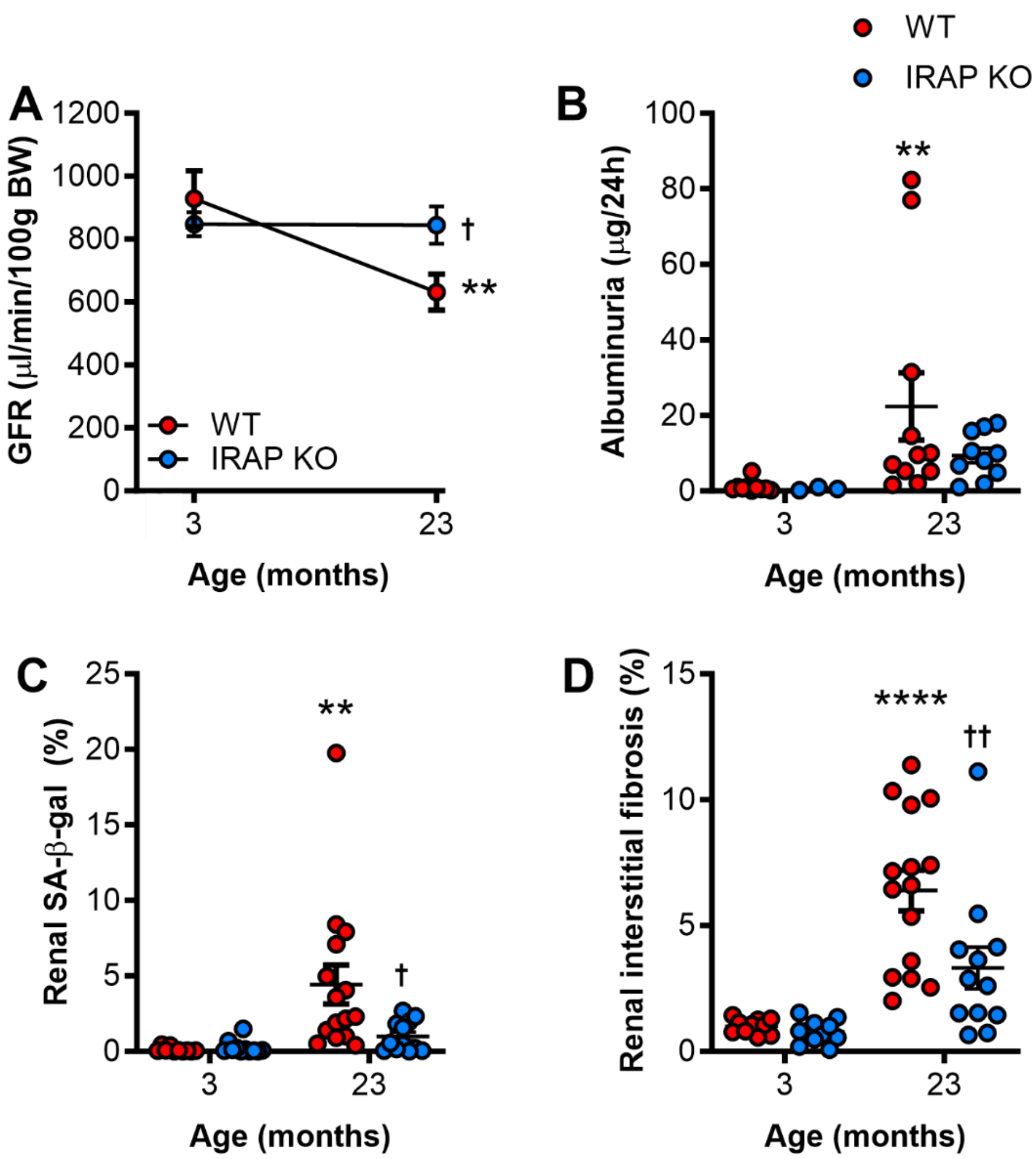

Figure S2. IRAP deficiency prevents age-related decline in renal function and accumulation of senescent cells in female mice.

(A) Glomerular filtration rate (GFR), (B) albuminuria, (C) renal senescence-associated β-galactosidase (SA-β-gal) staining and (D) Masson's trichrome staining to detect renal interstitial fibrosis in WT (open bars/points) and IRAP KO (closed bars/points) female mice from 3-23 months of age. N=10-15/group. Data are analysed via two-way ANOVA. *P<0.05, **P<0.01, ****P<0.0001 vs 3Mo WT; ††P<0.01 vs age-matched WT via a Sidak's multiple comparisons test.

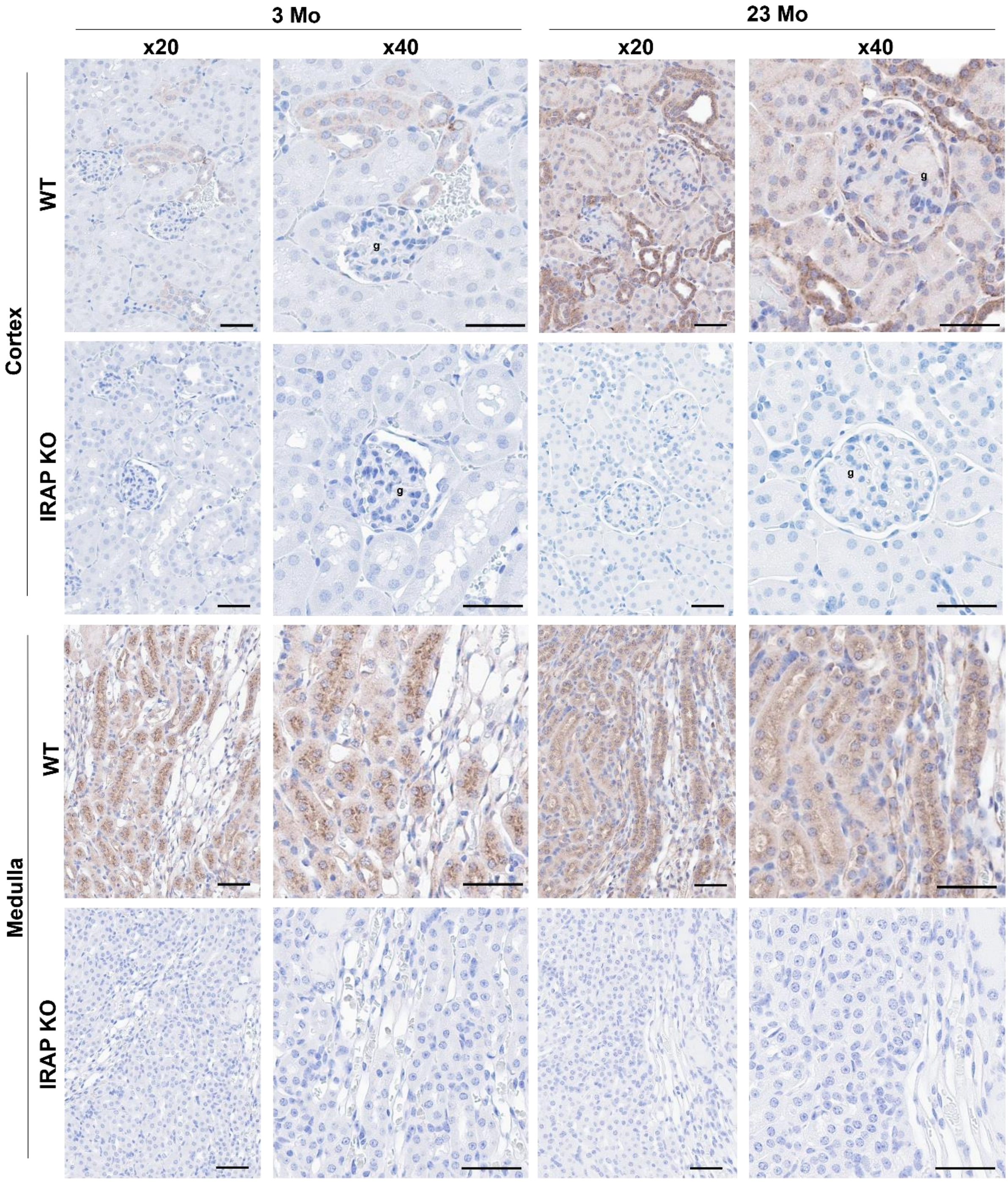

Figure S3. IRAP is expressed in the mouse kidney and is upregulated in ageing.

IRAP immuno-localisation in 3-month-old (Mo) wildtype (WT) and IRAP knockout (KO) mice. Scale=50 µM. g=glomerulus. IRAP expression was not detected in IRAP KO animals.

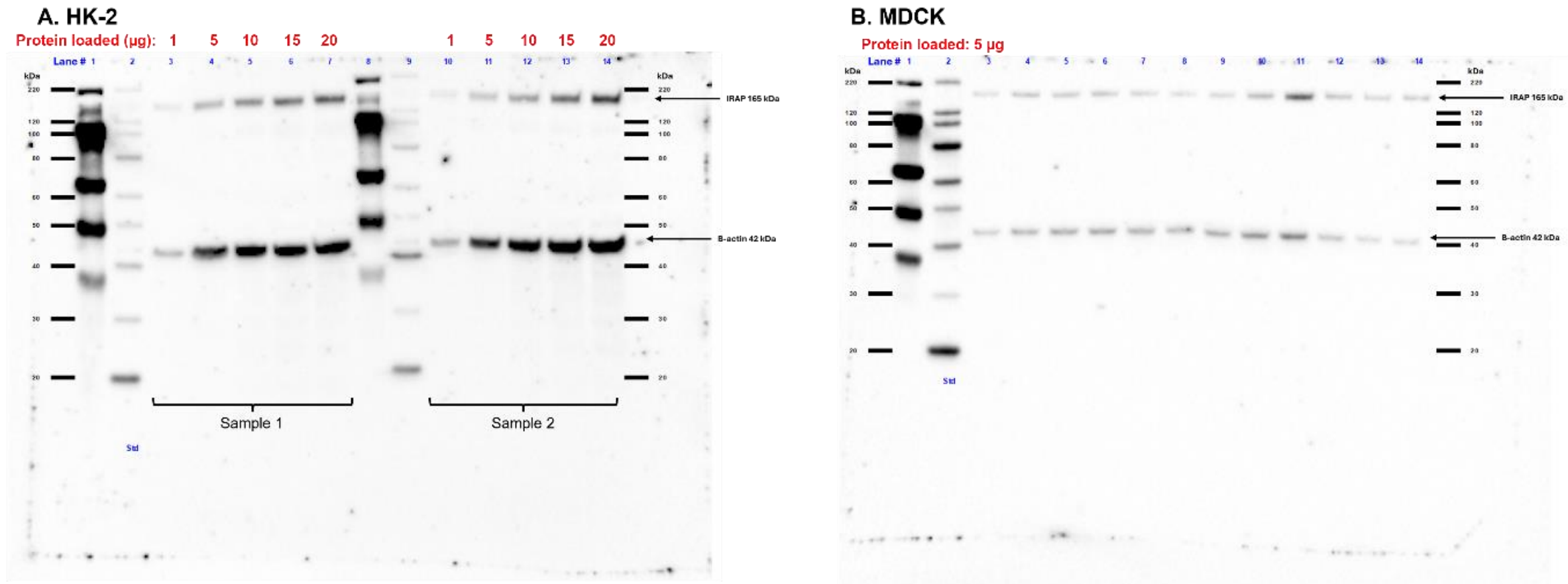

Figure S4. IRAP is expressed in HK-2 and MDCK cell lines.

Immunoblots showing the detection of IRAP protein (165 kDa) and B-actin (42 kDa) in (A) HK-2 and (B) MDCK cell lines. Optimal protein loading (1- 5 µg/well) was determined in two HK-2 samples, with 5 µg protein/well selected for MDCK blotting. HK-2: Lane 1 & 8 = SeeBlue Plus colorimetric ladder; lane 2 & 9 = MagicMark XP protein standard (Std). MDCK: Lane 1 = SeeBlue Plus colorimetric ladder; lane 2 = MagicMark XP protein standard.
